# Promoter DNA Methylation of *Oncostatin M receptor-β* as a Novel Diagnostic and Therapeutic Marker in Colon Cancer

**DOI:** 10.1371/journal.pone.0006555

**Published:** 2009-08-07

**Authors:** Myoung Sook Kim, Joost Louwagie, Beatriz Carvalho, Jochim S. Terhaar sive Droste, Hannah Lui Park, Young Kwang Chae, Keishi Yamashita, Junwei Liu, Kimberly Laskie Ostrow, Shizhang Ling, Rafael Guerrero-Preston, Semra Demokan, Zubeyde Yalniz, Nejat Dalay, Gerrit A. Meijer, Wim Van Criekinge, David Sidransky

**Affiliations:** 1 Department of Otolaryngology-Head and Neck Surgery, The Johns Hopkins School of Medicine, Baltimore, Maryland, United States of America; 2 OncoMethylome Sciences S.A, CHU Niveau +4Tour 4 dePharmacie (bâtiment 36), Liege, Belgium; 3 Department of Pathology, Free University of Amsterdam, Amsterdam, The Netherlands; 4 Department of Gastroenterology, Free University of Amsterdam, Amsterdam, The Netherlands; 5 Department of Medicine, Albert Einstein Medical Center, Philadelphia, Pennsylvania, United States of America; 6 Department of Pathology, The Johns Hopkins School of Medicine, Baltimore, Maryland, United States of America; 7 Oncology institute, Istanbul University, Istanbul, Turkey; Université Paris-Diderot, Paris 7, France

## Abstract

In addition to genetic changes, the occurrence of epigenetic alterations is associated with accumulation of both genetic and epigenetic events that promote the development and progression of human cancer. Previously, we reported a set of candidate genes that comprise part of the emerging “cancer methylome”. In the present study, we first tested 23 candidate genes for promoter methylation in a small number of primary colon tumor tissues and controls. Based on these results, we then examined the methylation frequency of *Oncostatin M receptor-β* (*OSMR*) in a larger number of tissue and stool DNA samples collected from colon cancer patients and controls. We found that *OSMR* was frequently methylated in primary colon cancer tissues (80%, 80/100), but not in normal tissues (4%, 4/100). Methylation of *OSMR* was also detected in stool DNA from colorectal cancer patients (38%, 26/69) (cut-off in TaqMan-MSP, 4). Detection of other methylated markers in stool DNA improved sensitivity with little effect on specificity. Promoter methylation mediated silencing of *OSMR* in cell lines, and CRC cells with low *OSMR* expression were resistant to growth inhibition by Oncostatin M. Our data provide a biologic rationale for silencing of *OSMR* in colon cancer progression and highlight a new therapeutic target in this disease. Moreover, detection and quantification of *OSMR* promoter methylation in fecal DNA is a highly specific diagnostic biomarker for CRC.

## Introduction

Colorectal cancer (CRC) is one of the most common cancers among men and women and accounts for 10% of all new cancer cases and cancer deaths each year [1]. The overall 5-year survival rate from colon cancer has increased during the past 20 years because of early detection from increased screening. In spite of much progress, more advanced knowledge of the molecular pathogenesis of CRC or key environmental/dietary factors in CRC development is still needed. Moreover, finding potential diagnostic markers and therapeutic targets for CRC will aid in the early detection and treatment of colon cancer. Most CRCs arise from adenomatous precursors, and accumulation of gain-of-function mutations in proto-oncogenes and loss-of-function mutations in tumor suppressor genes (TSGs) leads to progression of adenomatous lesions to carcinoma [2], [3], [4].

In addition to genetic alterations involving mutations of oncogenes and TSGs, carcinogenic progression from benign neoplasm to adenocarcinoma can occur through epigenetic changes in gene promoters [5]. Aberrant gene expression is a characteristic of human cancers, and changes in DNA methylation status can have profound effects on the expression of genes. TSGs display both genetic and epigenetic inactivation in human tumors, and the transcriptional silencing of TSGs has established hypermethylation as a common mechanism for loss of TSG function in human cancers [6] including colon cancer [7], [8]. Thus, knowledge of methylation patterns across the genome can help to identify key TSGs inactivated during tumor formation [9], [10], [11].

Previously, we reported a set of candidate genes that comprise part of the emerging “cancer methylome” by using a new promoter structure algorithm and microarray data generated from 22 cancer cell lines derived form 5 major cancer types [12]. In this earlier study, we examined newly identified cancer-specific methylated genes in a panel of 300 primary tumors representing 13 types of cancer. Based on the data, the number of known cancer-specific methylated genes was increased by approximately 40%.

In the present study, we re-examined the methylation status of cancer-specific methylated genes in a larger number of tissue and stool DNA from colon cancer patients as well as patients without cancer. We found that *Oncostatin M receptor-β* (*OSMR*) and *β-1,4-galactosyltransferase-1* (*B4GALT*) were highly methylated in primary CRC tissues but rarely in corresponding normal adjacent mucosa nor in non-malignant normal colon tissues. Methylation of these genes was also detected in stool DNA from colon cancer patients, but generally absent from non-cancer patients. Moreover, *OSMR* and *B4GALT* mRNA expression in colon cancer tissues was significantly down-regulated as compared to normal tissues. Functional studies revealed a suppressive role for *OSMR* in colon cancer progression. Hence, *OSMR* methylation is biologically relevant and can be frequently detected in primary CRC tumors and matched stool DNA.

## Results

Microarray analysis in three human colon cancer cell lines (HCT116, HT-29 and DLD1) identified 52 potential gene targets based on reactivation after treatment with 5-aza-dC [12]. Of these 52 genes, *sirtuin 2* (*SIRT2*) was counted twice in our candidate gene list because it has two different gene identification numbers (NM_012237 and NM_030593) in the NCBI database. In addition, *O6-methylguanine-DNA methyltransferase* (*MGMT*) was previously reported to harbor cancer-specific promoter methylation in multiple types of cancer including colon cancer [9], so we excluded *MGMT* from our further analysis (52 -2 = 50). *Secreted frizzled related protein 4* (*SFRP4*) was previously reported to be methylated in colon cancer [16], [17] and was included as a positive control for gene methylation in our study. *Neurotrophin tyrosine kinase receptor type 2* (*NTRK2*) and *urokinase type plasminogen activator* (*PLAU*) were also included because no reports on methylation of the two genes in colon cancer have been published yet. We thus examined the methylation status of a total of 50 genes by bisulfite-sequencing or C-MSP in colon cancer cell lines (HCT116, DLD1, RKO, and SW480) (Figure S1). As a result, we found 23 genes to be methylated in more than one cell line (Table 1). Methylation in the other 27 genes was not detected in any of the cell lines tested.

**Table 1:** Methylation profiles in CRC cell lines.

Refseq	Name	Method	HCT116	DLD1	RKO	SW480	% Frequency	
NM_015049	ALS2CR3	SEQ	U	U	U	U		U
NM_016201	AMOTL2	SEQ	U	U	U	U		U
NM_001677	ATP1B1	SEQ	U	U	U	U		U
NM_004323	BAG1	C-MSP	U	U	n/d	U		U
NM_001497	B4GALT1	C-MSP	M	M	M	M	100 (4/4)	M
NM_024834	C10orf119	SEQ	M	U	U	U	25 (1/4)	M
NM_003876	C17orf35	SEQ	U	U	U	U		U
NM_015578	C19orf13	SEQ	U	U	U	U		U
NM_001757	CBR1	SEQ	U	U	U	U		U
NM_004935	CDK5	SEQ	U	U	U	U		U
NM_016129	COPS4	SEQ	M	n/d	M	n/d	100 (2/2)	M
NM_006375	COVA1	SEQ	U	U	U	U		U
NM_004078	CSRP1	SEQ	M	n/d	M	M	100 (3/3)	M
NM_001349	DARS	SEQ	M	U	M	U	50 (2/4)	M
NM_018981	DNAJC10	SEQ	U	U	n/d	U		U
NM_017946	FKBP14	SEQ	M	M	M	M	100 (4/4)	M
NM_017739	FLJ20277	SEQ	M	M	n/d	M	100 (3/3)	M
NM_024619	FN3KRP	SEQ	M	M	M	M	100 (4/4)	M
NM_014610	GANAB	C-MSP	U	U	U	n/d		U
NM_015234	GPR116	SEQ	U	U	U	U		U
NM_000182	HADHA	SEQ	U	U	U	U		U
NM_004507	HUS1	SEQ	M	M	M	M	100 (4/4)	M
NM_003597	KLF11	SEQ	M	M	M	M	100 (4/4)	M
NM_018846	KLHL7	SEQ	U	U	U	U		U
NM_146388	MRPL4	SEQ	M	M	M	M	100 (4/4)	M
NM_002466	MYBL2	SEQ	M	M	n/d	M	100 (3/3)	M
NM_053030	MYLK	SEQ	U	M	M	M	75 (3/4)	M
NM_015537	NELF	SEQ	U	U	U	U		U
NM_018092	NETO2	SEQ	U	U	U	U		U
NM_006703	NUDT3	SEQ	U	U	U	U		U
NM_003999	OSMR	C-MSP	U	M	M	M	75 (3/4)	M
NM_004670	PAPSS2	SEQ, C-MSP	U	U	M	M	50 (2/4)	M
NM_006713	PC4	SEQ	U	U	U	U		U
NM_002898	RBMS2	SEQ	M	M	M	M	100 (4/4)	M
NM_015149	RGL1	C-MSP	U	U	n/d	U		U
NM_004040	RHOB	SEQ	U	U	U	n/d		U
NM_005505	SECTM1	SEQ	U	U	U	M	25 (1/4)	M
NM_003004	SCARB1	SEQ	U	U	n/d	U		U
NM_012237	SIRT2	SEQ	U	U	U	U		U
NM_016538	SIRT7	SEQ	M	M	M	M	100 (4/4)	M
NM_015139	SLC35D1	SEQ	U	U	U	U		U
NM_017767	SLC39A4	SEQ, C-MSP	M	M	M	M	100 (4/4)	M
NM_004252	SLC9A3R1	SEQ, C-MSP	M	M	M	M	100 (4/4)	M
NM_016614	TTRAP	SEQ	U	U	U	U		U
NM_016437	TUBG2	SEQ	M	M	M	M	100 (4/4)	M
NM_003345	UBE2I	SEQ	U	U	U	U		U
NM_130839	UBE3A	C-MSP	U	U	U	n/d		U
NM_006180	NTRK2	C-MSP	U	U	M	n/d	33 (1/3)	M
NM_002658	PLAU	C-MSP	U	U	U	n/d		U
NM_003014	SFRP4	C-MSP	M	M	M	n/d	100 (3/3)	M
NM_002412	MGMT							

When “methylation positive” was detected in any single cell line, it was considered as methylation (M) in cell lines.

No CpG islands in GPR116 and SLC39A4 were found within 1 kb upstream of the TSS.

C10orf119, SECTM1 and NTRK2 were only methylated in one cell line.

M, methylated; U, unmethylated. n/d, not determined.

**Figure 1:**
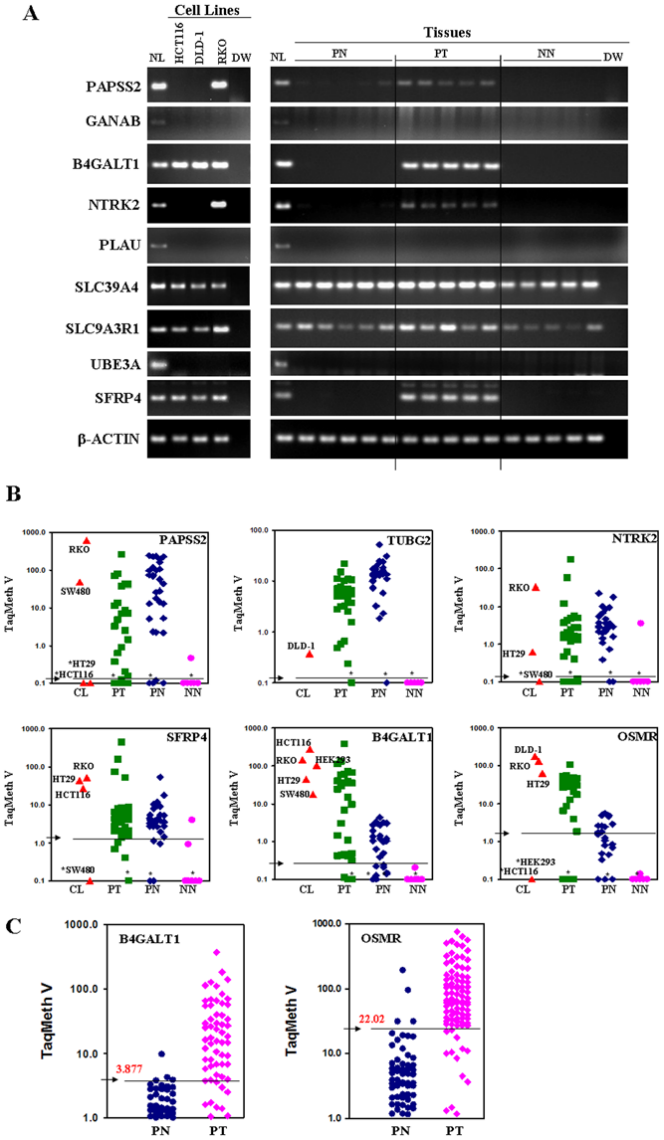
Promoter methylation analysis. A, Methylation status of each gene was examined in CRC cell lines and tissues by C-MSP after PCR with methylation-specific primers. PCR products were run on 4% agarose gels pre-stained with ethidium bromide. *In vitro* methylated, bisulfite-treated human normal lymphocyte DNA (NL) was used as a positive control for PCR, and distilled water (DW) was used as a negative PCR control. *β-actin* was used to confirm integrity of DNA. *SLC39A4* and *SLC9A3R1* were methylated both in cell lines and tissues, but *GANAB*, *PLAU* and *UBE3A* were not. The other 5 genes were methylated in one of three cells lines tested, and only methylated in CRC tissues (PT). PN, paired normal colon tissues from colon cancer patients; PT, paired CRC; NN, normal colon epithelium from non-cancer patients. C-MSP results of *OSMR* in CRC cell lines and tissues were shown in a previous report [12]. B, Scatter plots of methylation values of six candidate genes in tissues and cell lines (CL). The overall methylation values (TaqMan methylation values, TaqMeth V) and the optimal specificity/sensitivity at optimal cut-offs calculated from ROC analysis are shown in Table 3. Arrows indicate the optimal cut-off values for each gene. No cases of NN displayed TaqMeth V over the optimal cut-offs for *B4GALT1* and *OSMR*. HEK293, a non-tumorigenic cell line, harbored a high level of *B4GALT1* methylation (TaqMeth V, 101.12), but *OSMR* methylation was not detected in the cell line (TaqMeth V, 0.00). *, Samples with a ratio equal to zero could not be plotted correctly on a log scale, so are presented here as 0.1. All assays were performed in duplicate format, and experiments were repeated twice. Data showed reproducible and concordant results in triplicate. TaqMeth V is described in Materials and Methods. C, Quantitative methylation levels of *B4GALT1* and *OSMR* in primary colon tissues. Scatter plot of *B4GALT1* and *OSMR* promoter methylation. Arrows indicate optimal cut-off values for each gene (3.877 for *B4GALT1* and 22.01 for *OSMR*).

To identify genes harboring cancer-specific promoter methylation, we examined the methylation status of the 23 genes in five to ten pairs of matched primary CRC (PT) and corresponding normal colon (PN) tissues. Normal colon mucosa tissues from non-cancer patients (NN) were also included to compare methylation specificity between cancer and non-cancer patients. We excluded genes that harbored methylation in NN with frequencies higher than 30%. As a result, we found that *3′-phosphoadenosine 5′-phosphosulfate synthase 2* (*PAPSS2*), *γ-tubulin gene 2* (*TUBG2*), *NTRK2*, *B4GALT1*, and *OSMR* as well as *SFRP4* harbored cancer-specific methylation with high frequencies (>30%) (Table 2). All 6 of these genes did not harbor methylation in all NN tested, and differences in NN *vs.* PT and methylation *vs.* unmethylation cases were statistically significant. Representative results of C-MSP, bisulfite-sequencing, and COBRA in cell lines and tissues are shown in Figure 1A and Figure S2.

**Table 2:** Methylation profiles in colon tissues.

			% Frequency		*Fisher's exact*	
Genes	Method	PT	PN	NN	a *p value*	b *p value*
B4GALT1	C-MSP	100 (5/5)	0 (0/5)	0 (0/5)	*0.008* *	*0.008* * √
C10orf119	SEQ	0 (0/10)	0 (0/10)	0/10	*n/a*	*n/a*
COPS4	SEQ	0 (0/5)	0 (0/5)	0 (0/5)	*n/a*	*n/a*
CSRP1	SEQ	50 (3/6)	90 (9/10)	100 (5/5)	*0.118*	*0.182*
DARS	SEQ	22 (2/9)	40 (4/10)	40 (4/10)	*0.628*	*0.628*
FKBP14	SEQ	0 (0/5)	0 (0/5)	0 (0/4)	*n/a*	*n/a*
FN3KRP	SEQ	100 (5/5)	100 (5/5)	100 (5/5)	*n/a*	*n/a*
FLJ20277	SEQ	100 (5/5)	100 (5/5)	100 (5/5)	*n/a*	*n/a*
HUS1	SEQ	22 (2/9)	0 (0/5)	0 (0/5)	*0.505*	*0.505*
KLF11	SEQ	100 (6/6)	100 (6/6)	100 (6/6)	*n/a*	*n/a*
MYBL2	SEQ	100 (9/9)	70 (7/10)	100 (5/5)	*0.6*	*n/a*
MRPL4	SEQ	100 (5/5)	100 (5/5)	100 (5/5)	*n/a*	*n/a*
MYLK	SEQ	10 (1/10)	0 (0/10)	0 (0/10)	*1.000*	*1.000*
OSMR	C-MSP	100 (5/5)	33 (1/3)	0 (0/5)	*0.107*	*0.008* * √
PAPSS2	SEQ, C-MSP	100 (5/5)	20 (1/5)	0 (0/5)	*0.048* *	*0.008* * √
RBMS2	SEQ	100 (10/10)	100 (7/7)	100 (10/10)	*n/a*	*n/a*
SECTM1	SEQ	0 (0/5)	0 (0/5)	0 (0/5)	*n/a*	*n/a*
SIRT7	SEQ	100 (4/4)	100 (5/5)	100 (3/3)	*n/a*	*n/a*
SLC39A4	SEQ, C-MSP	67 (6/9)	100 (6/6)	100 (10/10)	*0.229*	*0.087*
SLC9A3R1	C-MSP	100 (5/5)	100 (5/5)	100 (5/5)	*n/a*	*n/a*
TUBG2	SEQ, C-MSP	71 (5/7)	40 (2/5)	0 (0/5)	*0.558*	*0.028* * √
NTRK2	C-MSP	100 (5/5)	20 (1/5)	0 (0/5)	*0.048* *	*0.008* * √
SFRP4	C-MSP	100 (5/5)	0 (0/5)	0 (0/5)	*0.008* *	*0.008* * √

PN, corresponding normal colon mucosa from CRC patients; PT, CRC tissues; NN, colon normal epithelium from non-cancer patients.

*P* value was calculated from the *Fisher's exact test.*

a PT vs. PN.

b PT vs. NN.

* P<0.05 was considered significant.

√ Determination of genes harboring cancer-specific methylation was based on *p<0.05* in PT *vs.* NN.

*n/a*, not assessed.

To study promoter methylation of these 6 genes by TaqMan-MSP real-time analysis, we designed primers and probes specifically targeting the CpG islands of each gene (Figure S3). We increased the sample numbers to over 25 pairs of primary CRC (PT) and corresponding normal colon (PN) tissues, and to 13 normal colon mucosa tissues from non-cancer patients (NN). The distribution of methylation values for each gene is shown in Figure 1B. Due to heterogenous clonal patches known to expand beyond the tumor borders, a low level of methylation in PN was also commonly observed. The overall methylation values (TaqMan methylation values, TaqMeth V) are shown in Table 3. *B4GALT1* and *OSMR* harbored higher levels of overall methylation in PT than those in PN and NN, and the differences were significant for both genes between PT and PN (*P<0.001*) and between PT and NN (*P<0.001*). When methylation values were compared within individual pairs of PN and PT samples, significantly higher methylation levels of *PAPSS2 TUBG2*, *NTRK2*, and *SFRP4* were found in 60% (18/30), 50% (15/30), 30% (9/30), and 36% (11/30), respectively, in PN than in corresponding tumor samples (PT). Higher methylation levels of *B4GALT1* in PN samples was found only in 4 cases (13.3%, 4/30), and methylation of *OSMR* was not found in any of the paired normals (0%, 0/25).

**Table 3:** Sensitivity and specificity of gene methylation at optimal cut-off values for detection of colon cancer tissue.

		TaqMeth V			[Table-fn nt117]			Optimal			
Gene	PT	PN	NN	a *P*	*^b^* *P*	AUROC	c *P*	Cut-off	% Specificity	% Sensitivity	d *P*
PAPSS2	19.466±49.251	71.528±81.698	0.035±0.126	*<0.001* *	*<0.001* *	0.7667±0.068	*<0.001*	0.121	92.3 (12/13)	70.0 (21/30)	*<0.001*
TUBG2	5.835±4.707	13.981±10.476	0.009±0.028	*<0.001* *	*<0.001* *	0.9667±0.033	*<0.001*	0.116	100 (13/13)	96.6 (29/30)	*<0.001*
NTRK2	9.647±32.432	4.185±5.220	0.278±1.002	*0.171*	*<0.001* *	0.8667±0.060	*<0.001*	0.118	92.3 (12/13)	83.3 (25/30)	*<0.001*
SFRP4	26.595±83.428	6.356±9.786	0.376±1.112	*0.991*	*<0.001* *	0.9397±0.042	*<0.001*	1.027	92.3 (12/13)	90.0 (27/30)	*<0.001*
B4GALT1	36.740±71.673	1.387±0.009	0.015±0.056	*<0.001* *	*<0.001* *	0.9256±0.034	*<0.001*	0.317	100 (13/13)	83.3 (25/30)	*<0.001*
OSMR	25.584±24.765	1.666±1.742	0.022±0.044	*<0.001* *	*<0.001* *	0.8846±0.044	*<0.001*	1.830	100 (13/13)	80.0 (20/25)	*<0.001*

TaqMeth V is expressed as mean±SD, and TaqMeth V is described in Materials and Methods.

P value was derived from Wilcoxon matched-pairs signed-ranks test.

a PT *vs.* PN.

* *P<0.01* was considered significant.

*P* value was derived from the *Wilcoxon-Mann-Whitney* test.

b PT *vs.* NN.

AUROC is expressed as mean±SD, and optimal cut-off values were calculated from ROC analysis.

c *P* value in ROC analysis (PT *vs.* NN).

d *P* value in *Fisher's exact* test (PT *vs.* NN).

Sensitivity, positive methylation/total tumor cases (PT); Specificity, negative methylation/total normal cases (NN).

% Methylation in PN was based on cut-offs from PT *vs.* NN.

Methylation level below the cut-offs was considered as unmethylated and over the cut-offs were as methylated.

PN, corresponding normal colon mucosa from CRC patients; PT, CRC tissues; NN, colon normal epithelium from non-cancer patients.

Methylation of the 6 genes in tissue showed highly discriminative receiver–operator characteristic (ROC) curve profiles, clearly distinguishing CRC (PT) from normal colon mucosa (NN) (*P<0.001*) (Figure S4A). AUROC (Area under ROC) was over 0.76 in all genes tested. In order to maximize sensitivity and specificity, the optimal cut-offs for the 6 genes were calculated from the ROC analysis (PT *vs*. NN) and are shown in Table 3. At cut-offs set for optimal specificity of 90% or more, the sensitivity of each gene in PT was over 70%. No cases of NN displayed TaqMeth V over the optimal cut-offs for *B4GALT1* and *OSMR* (100% specificity). The sensitivities of *B4GALT1* and *OSMR* were 83% (25/30) and 80% (20/25), respectively. These results indicate that *B4GALT1* and *OSMR* harbor cancer-specific methylation in CRC with high frequency. Therefore, we focused on *B4GALT1* and *OSMR* for further study.

We examined the methylation status of *B4GALT1* and *OSMR* in 100 new pairs of CRC (PT) and corresponding normal (PN) tissues by TaqMan-MSP analysis (Fig. 1C). The TaqMeth V in PT ranged from 0 to 370.70 (median value 3.70) for *B4GALT1* and from 0 to 749.27 (median value 59.24) for *OSMR*. The value in PN ranged from 0 to 9.70 (median value 0.26) for *B4GALT1* and to 190.68 (median value 1.54) for *OSMR*. The overall methylation levels of *B4GALT1* and *OSMR* detected in PT (22.33±48.69 for *B4GALT1* and 116.83±151.52 for *OSMR*, mean±SD, n = 100) were also significantly higher than those in PN (0.90±1.37 for *B4GALT1* and 6.49±21.52 for *OSMR*, mean±SD, n = 100) (*P<0.001*). At the optimal cut-offs (values, 3.87 for *B4GALT1* and 22.01 for *OSMR*) calculated from the ROC analysis (PT *vs*. PN) (Figure S4B), the specificity of the two genes was over 96%. The sensitivities of *B4GALT1* and *OSMR* were 49% (49/100) and 80% (80/100), respectively. Taken together, *B4GALT1* and *OSMR* were frequently methylated in primary CRC tissues but displayed absent or low levels of methylation in corresponding normal tissues.

**Figure 2:**
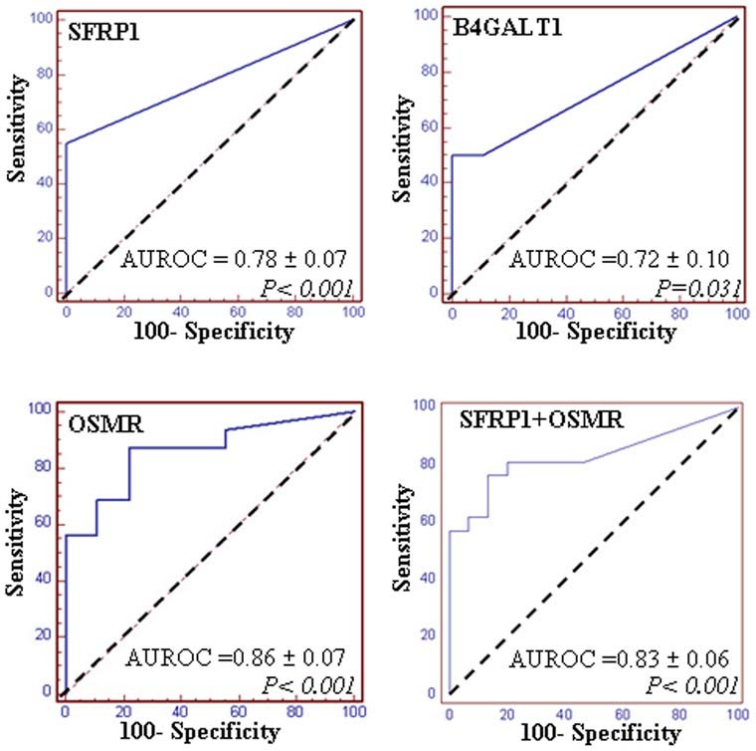
ROC curve analysis in stool DNA. A blinded test was performed for gene methylation analysis in stool DNA collected from patients with or without colon cancer. *SFRP1* was included to compare methylation frequency of a known cancer-specific methylated gene. Sensitivity/specificity based on optimal cut-offs calculated from ROC analysis is shown in Table 4.

Next, we performed a blinded analysis of gene methylation analysis in stool DNA collected from patients with or without colon cancer. The clinical status of the patients was not revealed until the analysis was completed. We also examined hypermethylation of *SFRP1* as a positive control for detection of gene methylation in stool DNA [18]. The results of ROC analysis in the stool DNA are shown in Figure 2. Table 4 shows the sensitivity/specificity of each gene for colon cancer detected in stool DNA samples. *SFRP1* methylation was not detected in patients with endoscopically normal colon (0%, 0/15), and was found in 55% (11/20) of CRC patients, at the optimal cut-off value calculated from the ROC analysis. Methylation of *B4GALT1* and *OSMR* was detected in 64% (9/14) and 80% (16/20) of stool DNA samples from CRC patients, respectively. When methylation of *SFRP1* and *OSMR* was combined, the sensitivity was 60% (12/20), and the specificity was 100% (0/15). Discrimination between patients without cancer and CRC patients by *SFRP1* or *OSMR* methylation was statistically significant (*P<0.01*).

**Table 4:** Gene methylation detected in stool from colon cancer or non-cancer patients.

	SFRP1			B4GALT1			OSMR			SFRP1+OSMR^d^		
Clinical features	M (+)	%	*P value*	M (+)	%	*P value*	M (+)	%	*P value*	M (+)	%	*P value*
Non-CRC/Non-AD^a^	0/15	0	^e^0.001*	2/10	20	*^e^0.109*	0/15	0	^e^0.004*	0/15	0	^e^<0.001*
CRC^b^	11/20	55		9/16	56		9/20	45		12/20	60	
AD^c^	5/17	29	*^f^0.185*		n/a		2/16	13	*^f^0.067*	6/17	35	*^f^0.191*

Methylation positivity and negativity based on methylation level at cut-off values; 0 for SFRP1, 0 for B4GALT1 and 8.53 for OSMR. M(+), Methylation positive.

a Patients with endoscopically normal colon (diverticulosis, diarrhea, polyps or bloody stool).

b Patients with colorectal cancer.

c Patients with adenoma.

d Sum of methylation from SFRP1 and OSMR. Sum of methylation positivity was calculated by counting cases determined to be methylation-positive for at least one gene.

Sum of methylation negativity was calculated by counting cases determined to be methylation-negative for each gene.

*Fisher's exact* test was performed in ^e^normal *vs.*CRC, and ^f^CRC *vs.* AD; *P<0.05 was considered significant. n/a, not assesed.

*OSMR* methylation was tested in a blinded fashion in one more independent set of stool samples (no overlap with the samples in Table 4). Stool DNA from healthy control subjects who had no visual abnormalties in colonoscopy were included for this study. The sensitivity of *OSMR* methylation in CRC patients was 38% (26/69) and the specificity was 95% (77/81) (Table 5). The difference between CRC patients and normal controls was statistically highly significant (*P<0.001*). In addition, *OSMR* methylation was detected in 56% (15/27) and 44% (8/18) stool DNA samples from CRC stage II and III patients, respectively. Moreover, 34 of 41 cases (83%) of fecal DNA from confounding control patients without CRC were completely negative for *OSMR* methylation. Other clinical parameters in confounding controls such as diverticulosis, hemorrhoid, and polyps were not significantly associated with *OSMR* methylation status in stool (data not shown).

**Table 5:** OSMR methylation detected in stool samples.

Clinical features	M (+)	%	e *P value*	f *P value*
Controls^a^	4/81	5		
CRC^b^				
Total	26/69	38	<0.001*	
Stage^c^ I	2/18	11	*0.299*	
II	15/27	56	<0.001*	
III	8/18	44	<0.001*	
IV	1/6	17	*0.307*	
Confounding controls^d^	7/41	17		*0.031*

Methylation positivity and negativity based on methylation level of OSMR at a cut-off value 4.

M (+), Methylation positive.

a Healthy control subjects with no visual abnormalities in colonoscopy.

b Patients with colorectal cancer.

c I-IV, UICC stages.

d Patients without CRC (polyps, diverticulosis, and hemorrhoid)

e P values from *Fisher's exact* test performed in Controls *vs.*CRC. *P<0.05, significant.

f P value from *Fisher's exact* test between total CRC *vs.*Confounding controls.

**Figure 3:**
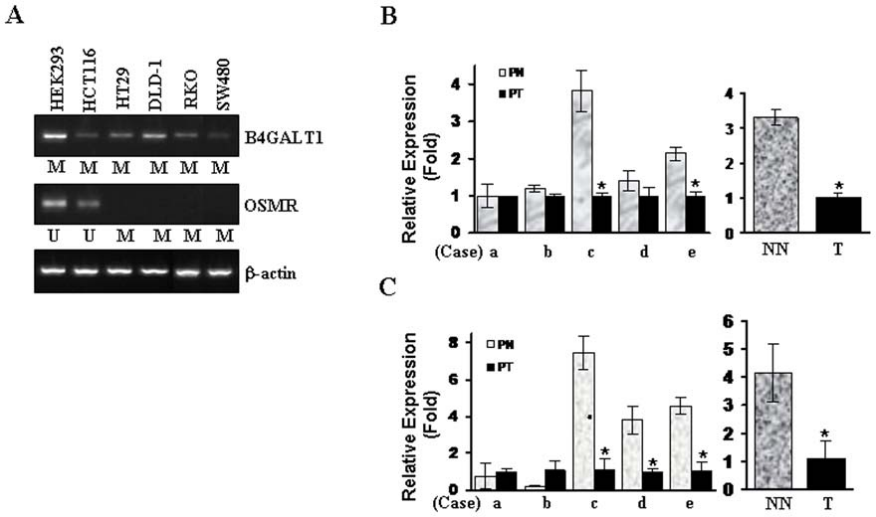
Expression of B4GALT1 and OSMR. A, Expression of *B4GALT1* and *OSMR* in cell lines was examined by RT-PCR analysis. Methylation of *B4GALT1* in the cell lines was examined by C-MSP or TaqMan-MSP. β-actin was used as a loading control. M, methylation; U, unmethylation. B and C, Real-time RT-PCR was performed in five pairs (a – e) of normal (PN) and tumor cDNA prepared from CRC patients (PT) (*left*). Expression of *B4GALT1* (B) and *OSMR* (C) were also compared between patients with colon cancer (T) or without cancer (NN) (*right*). Relative expression (Fold) was calculated by comparing the ratios of mRNA expression of *B4GALT1* and *OSMR* to an internal control gene, GAPDH. Experiments were done in duplicate, and values indicate means±SD. *, *P<0.05*. Experiments were done in duplicate, and values indicate means±SD. *, *P<0.05*.

To examine the transcriptional levels of *B4GALT1* and *OSMR*, we performed RT-PCR or Real-time RT-PCR analysis (Fig. 3) using cDNA prepared from CRC cell lines (HCT116, HT29, DLD-1, RKO, and SW480) and a non-tumorigenic cell line, HEK293. *OSMR* expression was observed only in the cells where methylation of *OSMR* was not found (HCT116 and HEK293). Increase of *OSMR* expression by 5-aza-dC treatment was previously reported [12], indicating that expression of *OSMR* correlates tightly with promoter methylation. Basal expression of *B4GALT1* was detected in all cell lines tested, and all of these cell lines also harbored methylation of *B4GALT1*. However, *B4GALT1* was further increased by 5-Aza-dC (Figure S5A), indicating that its expression was at least partially suppressed by promoter methylation.

**Figure 4:**
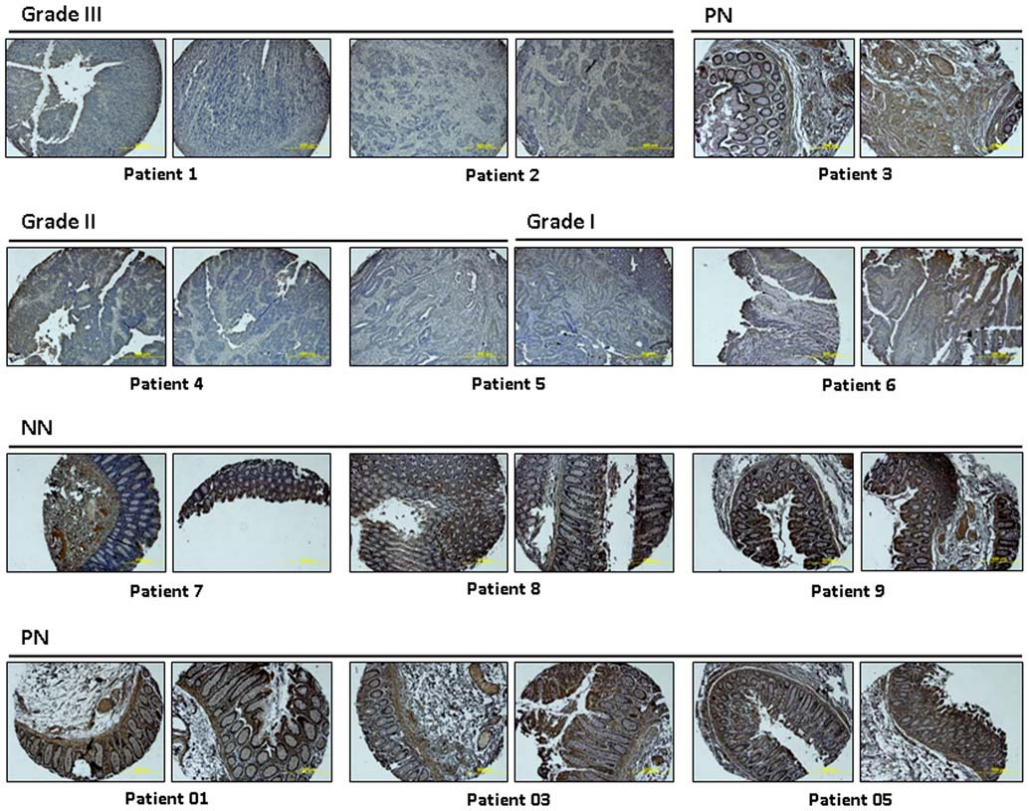
Immunohistochemical analysis of OSMR in colon cancer tissue microarray with normal colon tissue controls. Strong expression of *OSMR* was detected in all non-malignant normal tissues (NN) and adjacent normal colon mucosa (PN). In contrast, *OSMR* was rarely detected in the neoplastic cells from colon cancer patients. Tumor grades are indicated.

We then performed Real-time RT-PCR in cDNA prepared from tumor (PT) and corresponding normal tissues (PN) of five individual colon cancer patients (matched cDNA). In 3 of 5 tumor cases, *OSMR* was significantly down-regulated (Fig. 3C, *left*). The expression of *B4GALT1* and *OSMR* in tumor was three and four times lower than in normal tissue (NN), respectively (Fig. 3B and C, *right*). By immunohistochemical staining of a colon normal and cancer tissue microarray with an anti-OSMR antibody, we detected strong expression of *OSMR* in all non-malignant normal tissues (NN) and adjacent normal colon mucosa (PN) from colon cancer patients (Fig. 4 and Table 6). However, *OSMR* was barely detected in almost of all primary tumors (PT) (weak expression in 4 of 10 cases). These results suggest a specific decrease of *OSMR* mRNA and protein in colon cancer development.

**Table 6:** Immunohistochemical analysis of OSMR in colon cancer tissue microarray with normal tissue controls.

Tissues	Expression	Tumor Grade
**Colon Cancer**		
**1**	-	III
**2**	-	III
**3**	-	III
**4**	+	III
**5**	+	II
**6**	-	II
**7**	-	II
**8**	-	I
**9**	+	I
10	+	I
**Non-malignant normal colon**		
**1**	++	
**2**	++++	
**3**	++++	
**4**	++++	
**5**	++++	
**6**	++++	
**Cancer adjacent normal colon**		
**1**	++++	
**2**	++++	
**3**	++++	
**4**	++++	
**5**	++++	
**6**	++++	
**7**	++++	
**8**	++++	

Note, expression level is indicated as -, no or very faint expression.

+ mild expression

++ moderate expression.

+++ strong expression.

++++ very strong expression.

**Figure 5:**
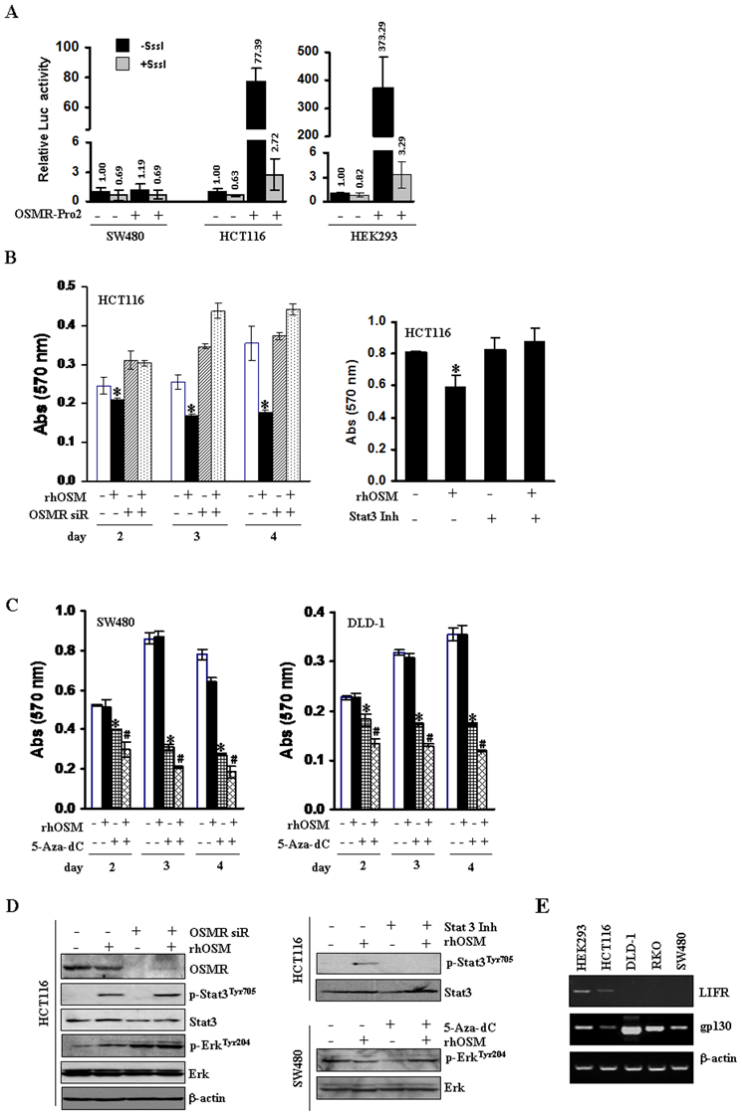
Cellular function of OSMR. A, Analysis of *OSMR* promoter activity by luciferase reporter assay in OSMR-positive (HCT116 and HEK293) and - negative (SW480) cells. The promoter construct was pre-treated with or without *Sss*I methylase for 8 hrs before transfection. The highest activity was detected in HEK293 cells. Data are expressed as fold increase over pGL3-basic activity. Experiments were done in triplicate, and values indicate means±SD. Mean values are presented. B, An siRNA pool targeting *OSMR* and non-targeting control were transiently transfected into HCT116, and cells were treated with rhOSM (50 ng/ml) (*left*). HCT116 cells were treated with or without Stat3 inhibitor peptide (Stat3 Inh, 100 μM) acting as a highly selective, potent blocker of Stat3 activation (*right*). Cell growth was determined by MTT assay. Data are expressed as absorbance at 570 nm. Experiments were done in triplicate, and values indicate means±SD. *, P<0.05. C, 5-Aza-dC (5 μM) was pre-treated for 48 hrs in SW480 and DLD-1 cells, and then co-treated with rhOSM for 48 hrs. Cell growth was determined by MTT assay. *, 5-Aza-dC-treated cells compared to untreated control (*P<0.05*); #, 5-Aza-dC/rhOSM- treated cells compared to 5-Aza-dC treatment alone (*P<0.05*). D, The phosphorylation of *Stat3* and *Erk* in response to rhOSM (50 ng/ml) was analyzed by Western blotting in HCT116 and SW480 cell lines. rhOSM increased phosphorylated *Stat3* and *Erk* in HCT116 cells but not in SW480 cells (which lack LIFR-see E below). Equal protein loading was monitored by total *Stat3*, *Erk* and *β-actin* evaluation in the samples. Re-activation of *OSMR* by 5-Aza-dC treatment was determined on SW480 and DLD-1 cells by flow cytometry (Figure S6). E. Expression of *LIFR* and *gp130* was examined in CRC cell lines by RT-PCR. *LIFR* was expressed in HCT116 cells but silenced in most other CRC cell lines tested. *gp130*, the *OSMR* heterodimer, was ubiquitously expressed in all cell lines tested. Promoter methylation status of these two latter genes was not examined.

To investigate the role of DNA methylation in regulation of *OSMR* expression, we transfected a pGL3-*OSMR*-Pro2-Luciferase construct into three cell lines; a *OSMR*-negative cell line, SW480, and two *OSMR*-positive cell lines, HCT116 and HEK293. The construct was treated with or without *Sss*I methylase before transfection. Activity of the *OSMR* promoter was not detected in SW480, but a high level of promoter activity was detected in HCT116 and HEK293 cells (Fig. 5A). Induction of CpG methylation with *Sss*I methylase decreased the activity to minimal levels.

Oncostatin M (OSM) is an interleukin-6 (IL-6)-type cytokine, but more active than IL-6 in inhibiting the proliferation of numerous solid tumor cell lines [19], [20]. Recently, a correlation of resistance to growth inhibition by OSM with specific loss of the *OSMR* and Stat3 signaling was reported [15]. In order to examine CRC cell resistance to growth inhibition by OSM, we transiently transfected a siRNA pool targeting OSMR and a non-targeting control siRNA into *OSMR*-expressing HCT116 cells, and performed a standard cell growth assay after treatment of cells with a recombinant human OSM (rhOSM). We observed significant growth inhibition by rhOSM in HCT116 cells, and the inhibition was reversed by knock-down of *OSMR* (Fig. 5B, left). A Stat3 inhibitor peptide (Stat3 Inh) that abrogated Stat3 activation (Fig. 5D) blocked the rhOSM-induced growth inhibition in HCT116 cells (Fig. 5B, right). Consistently, suppression of cell growth by rhOSM was not observed in SW480 and DLD-1 cells with *OSMR* promoter methylation (Fig. 5C). Interestingly, the inhibition of cell growth by 5-Aza-dC treatment in SW480 and DLD-1 cells (*, *P<0.05*) was significantly enhanced by the treatment of rhOSM (#, *P<0.05*). Finally we observed that rhOSM activated Stat3 phosphorylation in HCT116 cells, regardless OSMR expression levels (Fig. 5D, left). Expression of *gp130* and *LIFR* mRNA was observed in HCT116 cells (Fig. 5E), indicating that the activation of Stat3 in HCT116 cells with very low OSMR expression caused by siRNA transfection may be through gp130/LIFR (type I OSM receptor)-mediated signaling. Erk phosphorylation increased in HCT116 cells transfected with siRNA targeting *OSMR*, and the basal level of phospho-Erk in SW480 cells with *OSMR* methylation was higher than in HCT116 cells (Fig. 5D). 5-Aza-dC partially decreased activated Erk in SW480 cells, and rhOSM recovered basal Erk phopshorylation in the presence of 5-Aza-dC. Thus, methylated *OSMR* markedly decreased tumor-inhibiting signals from rhOSM and key downstream signaling events.

## Discussion

Promoter methylation of key regulatory genes drives the cancer process and in the right context can serve as a diagnostic marker and a therapeutic target. Cancer-specific methylation serves as an important biomarker for the early detection of cancer. Such markers may supplement the cytopathological assessment of tissue biopsies or potentially stand on their own as markers of disease in various bodily fluids such as stool. To this end, the methylation frequency of genes identified in primary tissues has important clinical implications.

A number of genes are commonly hypermethylated in colorectal cancer (CRC) including *hMLH1*, *p16INK4a*, *p14ARF*, *RAR-β*, *APC*, *MGMT*, *cyclin A1*, *CDX1*, *MYOD1*, *COX-2* and *WT-1* [21], [22], [23]. However, genes methylated only in neoplastic tissues with high frequency (over 40%) are rare. In this study, we identified *PAPSS2*, *TUBG2*, *NTRK2*, *B4GALT1* and *OSMR* [12], [24] as genes harboring cancer-specific promoter methylation in human colorectal cancer. The theoretical sensitivity of each methylated gene was over 70% and the specificities were over 90% by TaqMan-MSP. The methylation frequency of each gene ranks with only a few other genes methylated at high frequency in CRC (*Cyclin A1, CDX1, RAR-β, MYOD1, p15INK4b and COX-2*) in a cancer-specific manner [10].

Studies on *B4GALT1* and *OSMR* have been reported in human cancer. *B4GALT1* is localized both in the Golgi complex and on the cell surface [25], and is constitutively expressed in all tissues including human colorectal mucosa [26] with the exception of the brain [27]. The role of cell surface *B4GALT1* in human cancer has been reported; it is an estrogen-regulated gene in MCF-7 cells [28], and its level was altered in highly metastatic lung cancer cells compared with its less metastatic parental cells [29]. *B4GALT1* promotes apoptosis by inhibiting the epidermal growth factor receptor pathway [30] and increases cycloheximide-induced apoptosis in human hepatocarcinoma cells [31]. Protein kinase B/Akt inhibits apoptosis by down-regulation of *B4GALT1* [25]. In addition, enhanced epithelial cell proliferation of the skin and small intestine and abnormal differentiation in intestinal villi were found in *B4GALT1*-deficient mice [32], suggesting that *B4GALT1* plays an important and suppressive role in the proliferation of epithelial cells. Thus, its inactivation by promoter methylation could lead to escape of normal cellular controls and cancer progression.

OSMR is a receptor of Oncostatin M (OSM), an interleukin-6 (IL-6)-type cytokine identified as a potent suppressor of tumor cells. Human OSM was originally described by its capacity to inhibit melanoma proliferation in vitro [33], [34], and its targets for growth inhibition include lung carcinomas [35], ovarian carcinomas [36], and breast tumors [37]. Resistance to growth inhibition by OSM in metastatic melanoma cell lines correlated with a specific loss of OSMR, in conjunction with a lower level of histone acetylation in the OSMR promoter region, suggesting that metastatic melanoma cells could escape the growth control of OSM by the epigenetic silencing of OSMR [15]. We discovered that promoter methylation strongly correlates with OSMR expression and also found a correlation of resistance to growth inhibition by OSM with loss of OSMR in CRC cell lines. Thus, promoter methylation is a key regulator of OSMR expression and all of these results support a suppressive function for OSMR in human cancer.

Human OSM forms two types of heterodimeric signaling complexes; gp130/leukemia inhibitory factor receptor (LIFR) (type I OSM receptor complex) [39] and gp130/OSMR (type II OSM receptor complex) [40]. gp130/LIFR can be activated by LIF or OSM, but gp130/OSMR is activated by OSM only. The type II receptor complex activates OSM-specific signaling pathways via the JNK/SAPK and Stat1/Stat5 pathways, whereas both type I and type II complexes activate Stat3 and Erk as common signaling pathways in breast cancer cells [41]. In addition, type I and type II receptor signaling may exhibit antagonistic functions [42]. We found that OSM-mediated cell growth inhibition was not observed in HCT116 cells with low OSMR level despite Stat 3 phosphorylation. Since HCT116 cells expressed gp130 and LIFR, rhOSM likely phosphorylates Stat3 through type I OSM receptor-mediated signaling, but in the absence of gp130/OSMR this effect is not sufficient to mediate sustained growth suppression [41]. In support of this notion, rhOSM did not increase Stat3 phosphorylation in SW480 cells which lack LIFR expression

From a clinical point of view, our results have potential immediate diagnostic and therapeutic implications and deserve further attention. In a blinded test performed in stool DNA from CRC patients, *B4GALT1* and *OSMR* methylation were successfully detected with high frequency and thus have potential for identifying individuals with colon cancer. When we tested an additional known methylated gene, *SFRP1* [18], in combination with *OSMR* the sensitivity of the assay increased; 60% (12/20) of colon cancers were detected in stool DNA with perfect specificity (*P<0.001*). These data suggest that promoter methylation of *OSMR* might serve as a true indicator for the presence of colon cancer.

Therapeutically, both genes provide tantalizing clues for new approaches in the treatment of CRC. Since *B4GALT1* promotes apoptosis by inhibiting the epidermal growth factor receptor pathway [30], strategies to reverse promoter methylation and re-express the gene might augment treatment with antibodies against the EGFR receptor. Kras mutation is known to be a strong indicator of resistance to EGFR targeted therapies [42], [43]. It would be interesting to know how many wild type *K-ras* colorectal cancers harbor *B4GALT1* methylation as a mechanism of EGFR resistance. Demethylation of *OSMR* could also have therapeutic impact by re-sensitizing cells to the inhibitory effects of OSM. This approach could have an impact on multiple tumors types beyond CRC since so many tumors have been found to be inhibited by OSM. Finally, one could even envision a combined approach where the addition of demethylation agents and OSM to anti-EGFR antibodies could target colon cancer cells and greatly diminish their chance of therapeutic escape.

## Materials and Methods

### Cell lines and tissues

Five CRC cell lines (HCT116, DLD1, RKO, SW480 and HT29) were purchased from ATCC (Manassas, VA). CRC cell lines were grown in 5X McCoy medium supplemented with 10% fetal bovine serum. HEK293 cells were obtained from ATCC and were grown in DMEM supplemented with 10% FBS. One hundred pairs of gDNA from primary colorectal cancers (PT) and matched normal adjacent colon mucosa (PN) were described previously [13]. For statistical purposes, PT and PN were treated as paired groups. Thirteen normal colon epithelial tissues (NN) were obtained from patients without cancer from the Department of Pathology, The Johns Hopkins University. Stool gDNA from colon cancer patients, patients without cancer, and healthy control subjects were kindly provided by OncoMethylome Sciences (Sart-Tilman, Belgium). Written informed consent was obtained from the patients who provided the colon epithelial tissues and the stool gDNA, and this study was approved by the Institutional Review Board of the Johns Hopkins University in US and Vrije Universiteit Medisch Centrum in Belgium.

### DNA purification from stool DNA and bisulfite treatment

Stool specimens were collected and immediately submerged in stool stabilization buffer (Exact Sciences, MA) and stored at room temperature until processing (within 72 hrs). For recovery of human DNA, whole samples were homogenized in excess volume (1∶7) of stool homogenization buffer (Exact Sciences, MA) and aliquoted in portions of 32 ml (the equivalent of 4 g of stool). Each aliquot of stool samples were centrifuged and the supernatants were incubated with RNase A for 1 hr at 37°C. The DNA was then precipitated with sodium acetate (pH 5.2) - isopropanol and washed with 70% ethanol. The DNA pellet was subsequently re-suspended in 4 ml of 1× TE (pH 7.4) and proteinase K digested by the use of 400 µl of 10X buffer (240 mM EDTA, 750 mM NaCl, pH 8.0), 400 µl of 10% SDS and 20 µl of proteinase K (20 mg/ml), and samples were incubated overnight at 48°C with constant shaking. After centrifugation, 5 ml of phenol/chloroform/isoamylalcohol was added and samples were incubated (with shaking at 225 rpm) for 10 min at RT. After centrifugation again, the aqueous layer was transferred into new tubes, and DNA was precipitated and washed. Pellets were re-suspended in 2 ml of 1× TE solution (pH 8.0). Bisulfite treatment of 1 µg of tissue gDNA was performed to convert unmethylated cytosines to uracils for methylation analysis. For stool DNA, an up-scaled DNA modification step was applied to 32 µg of the obtained DNA, using the EZ-96DNA Methylation Kit (Zymo Research, Los Angeles, CA), according to the manufacturer's protocol. Bisulfite-treated DNA was concentrated using the Clean and Concentrator Kit (Zymo Research) and eluted in 30 µl.

### Sequencing and Combined Bisulfite Restriction Analysis (COBRA)

All PCR reactions were done as described previously [14], and the primer sequences of bisulfite-DNA amplification were described previously [12]. PCR products were gel-extracted (Qiagen, Valencia, CA) and sequenced with an internal primer (F2) or forward primer (F1) using the ABI BigDye cycle sequencing kit (Applied Biosystems, Foster City, CA). Searches for CpG islands in each gene promoter were done by using the online accessible software Methprimer. Bisulfite-sequencing primers were designed at the CpG islands within 1 or 2 kb upstream of the transcription start site (TSS). For COBRA, eluted DNA after gel extraction was digested with *BstU1* (New England Biolabs., Beverly, MA), which recognizes the CGCG sequence, for 3 hrs at 60°C. Samples were loaded on a 10% acrylamide gel, stained with 1X SYBR Green Gold (Molecular Probes, Eugene, Oregon), and visualized under UV light.

### The criteria to determine methylation in cell lines and tissues

Bisulfite-sequencing was based on nucleotide sequences in electropherograms. When only a cytosine or a thymidine peak existed in a CpG, the sequence was “CG” (100% methylation) or “TG” (0% methylation). When both methylated and unmethylated alleles were observed in a CpG sequence, it was considered as “partially methylated” (M/U). When “partial methylated CpG” was observed, a cytosine peak was compared to a thymidine peak in the CpG. If a cytosine peak was similar to a thymidine peak or dominant, the sequence in electropherograms was “NG” or “CG”, indicating that over 50% methylated alleles existed. When a thymidine peak was dominant, the sequence was “TG”, indicating less than 50% methylated alleles. Only “NG” and “CG” were considered as “methylated” in the CpG. When “methylated” CpG was found in more than 50% of total CpGs in an amplified PCR product, it was considered as “methylation-positive.” When any CRC cell line was “methylation-positive,” it was classified as “methylation”. Methylation in tissues was determined when “methylation-positive” cases were observed in over 30% of total tissues tested (over 30% frequency).

### Conventional methylation-specific PCR (C-MSP)

Bisulfite-treated DNA was amplified with either methylation-specific or unmethylation-specific primers for each gene. Primer sequences are shown in Table S1. PCR reactions were performed for 35 cycles of 95°C for 30 sec, 58°C for 30 sec, and 72°C for 30 sec. When clear PCR products amplified with methylation-specific primers were detected, it was considered as “methylation-positive.” Determination of overall methylation in cell lines and tissues were the same as described above.

### Quantitative methylation-specific PCR (TaqMan-MSP)

For quantitative methylation analysis, PCR primers were designed to hybridize to the region of each gene that was determined to be methylated in CRC cell lines by bisulfite-sequencing or C-MSP, and a fluorescent probe was synthesized to the amplified region of the DNA. Primer and probe sequences for TaqMan-MSP are shown in Table S2. All oligonucleotide primer pairs were purchased from Invitrogen (Carlsbad, CA), and the TaqMan probe from VWR (West Chester, PA). All protocols for TaqMan-MSP were performed as reported [14], and all reactions were performed in duplicate. To ensure the specificity of the TaqMan-MSP analysis, each 384-well PCR plate had wells that contained bisulfite-converted DNA isolated from patient tissue samples and wells that contained the following controls: *in vitro* methylated normal lymphocyte DNA (NL, positive control), DNA from normal colon mucosa in which each gene is not methylated (negative control), and multiple water blanks (control for PCR specificity). Lymphocyte DNA from a healthy individual was methylated *in vitro* with excess *SssI* methyltransferase (New England Biolabs Inc., Beverly, MA) to generate completely methylated DNA, and serial dilutions (90–0.009 ng) of the DNA were used to construct a calibration curve for each plate. All samples were within the assay's range of sensitivity and reproducibility was based on amplification of an internal reference standard (threshold cycle [CT] value for β-actin of≤40). The methylation ratio (TaqMan methylation value, TaqMeth V) was defined as the quantity of fluorescence intensity derived from promoter amplification of each gene divided by fluorescence intensity from β-actin amplification, and multiplied by 100 (*TUBG2, NTRK2, SFRP4, and OSMR*) or 1000 (*B4GALT1* and *PAPSS2*). This ratio was used as a measure for the relative level of methylated DNA in samples. The samples were categorized as unmethylated or methylated based on optimal cut-offs from ROC analysis.

### Statistical Analysis

We used gene methylation levels (TaqMeth V) to construct receiver operating characteristic (ROC) curves for the detection of colon cancer. In the ROC analysis, tangent points where the slopes of ROC curves were 1.00 have been selected as optimal cut-off points to balance sensitivity and specificity. P value was derived from Z value that was calculated from the equation of (AUROC-0.5)/Std Err (standard error of AUROC). The cut-off values determined from ROC curves were then applied to determine the frequency of gene methylation. Samples with a methylation level higher than cut-offs were designated as methylated, and samples with a methylation level lower than cut-offs were designated as unmethylated. All Statistical analyses in this study were conducted using STATA Version 9 (STATA Inc., College Station, TX).

### 5-Aza-dC treatment, RT-PCR and Real-time RT-PCR

Cells were treated with 5 μM 5-aza-2′-deoxycytidine (5-Aza-dC) (Sigma, St. Louis, MO) every 24 hrs for 3 days. RNA was extracted using Trizol (Invitrogen, Carlsbad, CA) and reverse-transcribed with Superscript II reverse transcriptase (Invitrogen). RT-PCR was performed by 30 cycles of 95°C for 1 min, 58°C for 1 min, and 72°C for 1 min. PCR products were gel-extracted and sequenced to verify true expression of the genes. Five matched normal and tumor cDNA (a-e) were purchased from Clontech Laboratories, Inc. (Mountain View, CA), and cDNA panels of human normal colon tissue (NN) and colon cancer tissue (T) were purchased from BioChain Institute, Inc. (Hayward, CA). One μl of each cDNA was used for real-time RT-PCR using QuantiFast SYBR Green PCR Kit (Promega, Valencia, CA). Amplifications were carried out in 384-well plates in a 7900 Sequence Detector System (Perkin-Elmer Applied Biosystems, Norwalk, CT). Thermal cycling was initiated with a first denaturation step at 95°C for 3 minutes, followed by 40 cycles of 95°C for 15 seconds, 58°C for 30 seconds, 72°C for 30 seconds. Expression of genes relative to GAPDH was calculated based on the threshold cycle (C_t_) as 2^−Δ(ΔCt)^, where ΔC_t_ = C_t,GENE_−C_t,GAPDH_ and Δ (ΔC_t_) = ΔC_t,N_−ΔC_t,T_ (N, matched normal tissue cDNA; T, tumor tissue cDNA; NN, normal tissue cDNA from patients without cancer). Primer sequences are shown in Table S3.

### Immunohistochemistry

Tissue microarrays were performed with sections (5 µm) of colon cancer tissues, adjacent tissues 1.5 cm away from tumor, and non-malignant normal colon tissues which were purchased from US Biomax, Inc. (Rockville, MD). The tissues were deparaffinized and incubated with anti-*OSMR* rabbit polyclonal antibody (1∶100 dilution) (Santa Cruz Biotechnology, Santa Cruz, CA) at 4°C overnight. They were then incubated in broad spectrum secondary antibody purchased from DAKO (Carpinteria, CA) for 30 min. After washing the slides in PBS, tissue sections were stained with freshly prepared DAB chromogen solution (DAKO). We treated tissues with streptavidin and biotin (Invitrogen) for 20 min each to block endogenous biotin levels. Sections were counterstained in Mayer's Hematoxyline.

### Luciferase reporter assay

The pGL3-*OSMR*-Pro2-Luciferase construct designed to contain a CpG island (−693 to+229 relative to the TSS) was kindly provided by Dr. Frederic Blanchard (Université de Nantes, France) [15], and transfected into HEK293, HCT116 and SW480 cells at a density of 1×10^5^/well in a 24-well plate. For each well, 100 ng of the pGL3-*OSMR*-Pro2-Luciferase constructs was co-transfected with 10 ng of internal control reporter pSV-*Renilla* (Promega) using Fugene-6 (Roche, Basel, Switzerland) in accordance with the manufacturer's instructions. After 48 hrs, the luciferase assay was performed using a Dual luciferase assay kit (Promega) and a single-sample luminometer (PerkinElmer, Waltham, MA). The luciferase activity was normalized by pSV-*Renilla* activity, and the pGL3–basic vector was used as a control. The pGL3-*OSMR*-Pro2-Luciferase construct and the pGL3–basic vector were methylated *in vitro* using *Sss*I (CpG) methylase as recommended by the manufacturer's instructions (New England Biolabs, Beverly, MA). After DNA isolation, equal amounts (100 ng) of the methylated or unmethylated luciferase constructs were transfected into cells. Each experiment was performed twice, each in triplicate.

### Knockdown of *OSMR* and cell growth assay

A siRNA pool targeting *OSMR* and non-targeting control were purchased from Dharmacon (Chicago, IL). Fifty nM of each siRNA were transiently transfected to HCT116 using LipofectamineRNAiMax transfection reagent (Invitrogen) in OPTI-MEM. After 24 hrs, cells were incubated in complete growth medium. Initial cell seeding density was 5×10^3^/well in 24 well plates, and the antiproliferative activity of rhOSM (50 ng/ml) (R & D systems, Minneapolis, MN) was measured by the MTT assay. After 4 hr incubation in serum-deprived condition, cells were treated with rhOSM in 0.1% serum medium, and incubated for 2, 3, or 4 days as indicated. Stat3 inhibitor peptide (100 μM, cell permeable) purchased from Calbiochem (La Jolla, CA) was pre-treated for 1 hr and 30 min during serum starvation, and co-treated with rhOSM for 72 hrs. 5-Aza-dC (5 μM) (Sigma, St. Louis, MO) was pre-treated for 48 hrs, and co-treated with rhOSM.

### Western blot analyses

After pre-treatment with 5-Aza-dC or Stat3 inhibitor, rhOSM was treated in serum-free condition for 30 min, and whole cell lysates were extracted in RIPA buffer, separated on 4–12% gradient SDS-PAGE, and transferred to nitrocellulose. The blots were incubated with either anti-*OSMR*, anti-phospho-*Erk* (Tyr204), anti-phospho-*Stat3* (Tyr705), anti-*Erk*, anti-*Stat3*, or anti*-β-actin* antibody for 2 hrs at room temperature or 4 °C overnight. After antibody washing, the blots were incubated with their respective secondary antibody and detected with enhanced chemiluminescence reagents (Amersham, Pittsburgh, PA) according to the supplier's protocol. All antibodies were purchased from Cell Signaling Technology (Danvers, MA) except anti-*OSMR* and anti-phospho-*Erk* (Tyr204) (Santa Cruz Biotechnology) and anti*-β-actin* (Sigma) antibodies.

### Flow cytometry analysis

Cell surface expression of *OSMR* on SW480 and DLD-1 cells was detected using an anti-*OSMR*-phycoethryin (PE) conjugated antibody (Santa Cruz Biotechnology). Mouse IgG-PE antibody was used as an isotype control (Santa Cruz Biotechnology). Cell-associated fluorescence was acquired by a FACScaliber fluorocytometer (BD Biosciences) and analyzed using Cell-Quest software.

## Supporting Information

Figure S1 Fifty-two candidate genes thought to be relevant to colon cancer by the new promoter structure algorithm were identified after initial analysis. A total of 50 genes were analyzed for detection of promoter methylation in CRC cell lines. UnM, unmethylated gene group; M, methylated gene group.
(0.11 MB PPT)

Figure S2 Representative results of bisulfite sequencing in CRC cell lines and tissues (A). Genomic and bisulfite-treated genomic DNA sequences are indicated. *, Cytosine that was not protected by methylation was thus converted to Thymidine after bisulfite treatment. Underlined CGs, methylated CpGs that were maintained after bisulfite treatment. Primer sequences for bisulfite-sequencing were described previously (12). B, Promoter methylation of TUBG2 in cell lines was examined by C-MSP (a), and in tissues was examined by COBRA after digestion of gel-extracted PCR products with BstU1 (b). Samples were loaded on a 10% acrylamide gel, stained with 1 X SYBR Green Gold (Invitrogen) and visualized under UV light. Multiple cleaved bands by BstU1 digestion were detected in PT samples (1–5) after gel separation, indicating the continued presence of protected CGCG sequences as a result of methylation, whereas no BstUI cleavage was found in any NN samples (6–10). Due to tissue heterogeneity, methylated and unmethylated alleles co-exist in PT samples so that uncleaved bands can be seen. Mock digestion (without BstU1) of PT samples resulted in the same uncleaved band as BstU1 digestion of the normal colon mucosa PCR product. PT, paired CRC; NN, normal colon epithelium from non-cancer patients. (c), Representative bisulfite-sequencing results of the TUBG2 promoter. Only methylated alleles were detected in all cell lines tested whereas methylated/unmethylated alleles coexisted in PT and PN tissues. The criteria to determine methylation in cell lines and tissues are described in Materials and Methods.
(1.48 MB PPT)

Figure S3 CpG islands 1 Kb upstream of the transcription start site (TSS) in the candidate gene promoters. Graphics depicting CpG islands (gray) were taken from Methprimer software. NTRK2 and OSMR had two CpG islands and the other four genes had one CpG island within 1 Kb upstream of the TSS. Promoter regions analyzed in this study partially or completely covered the CpG islands indicated. Methylation for PAPSS2, TUBG2 and OSMR was examined at two different promoter regions. The location of the TSS is shown in each gene promoter. Primer sites for bisulfite-DNA amplification and C-MSP and TaqMan-MSP are indicated as Bi-F and Bi-R, and mF and mR, respectively. Probes for TaqMan-MSP analysis are indicated as P. Primers for C-MSP (mF-mR) were used for TapMan-MSP analysis. F, forward; R, reverse.
(0.43 MB PPT)

Figure S4 A, ROC curve analysis of 6 candidate genes (PT vs. NN). Area under the ROC (AUROC) conveys the accuracy in distinguishing NN from PT in terms of sensitivity and specificity. Solid line, genes analyzed; dashed line, no discrimination. B, ROC curve analysis (PT vs. PN) of B4GALT1 (left) and OSMR (right) in 100 pairs of CRC (PT) and corresponding normal (PN) tissues.
(0.38 MB PPT)

Figure S5 Expression of B4GALT1 and OSMR. A, B4GALT1 was reactivated by the 5-Aza-dC treatment (Aza), and OSMR reactivation by 5-Aza-dC was previously reported [12]. Fold induction was calculated by comparing the ratios of B4GALT1 or OSMR mRNA to GAPDH (an internal control) before and after treatment. Fold induction ranged from 1.7 to 4.4 for B4GALT1 and from 5.2 (HCT116) to 2,868 (DLD-1) for OSMR (12). Expression of the B4GALT1 (B) and the OSMR (C) in CRC cell lines was quantitatively compared by real-time RT-PCR. The lowest expression of OSMR was detected in SW480 cell line. Experiments were done in duplicate, and values indicate means±SD. *, P<0.05.
(0.88 MB PPT)

Figure S6 Re-activation of OSMR by 5-Aza-dC treatment. Cell surface expression of OSMR was determined on SW480 and DLD-1 cells by flow cytometry. Values shown as insets correspond to the mean of fluorescent intensity ratios between cells with or without 5-Aza-dC treatment (3 days). Mouse IgG-PE antibody was used as an isotype control (data not shown).
(0.14 MB PPT)

Table S1 mF, methylation-specific forward; mR, methylation-specific reverse; uF, unmethylation-specific forward; uR, unmethylation-specific reverse. aPrimers used for detection of methylatoin in cell lines; bPimers in cell lines and tissues.
(0.19 MB PPT)

Table S2 (0.17 MB PPT)

Table S3 Primers for RT-PCR are the same as for Real-Time RT-PCR.
(0.11 MB PPT)
