# Supplementary material for: Promoter DNA Methylation of Oncostatin M receptor-β as a Novel Diagnostic and Therapeutic Marker in Colon Cancer
Source: PLoS One. 2009 Aug 7;4(8):e6555. doi: 10.1371/journal.pone.0006555 (PMC2717211; doi:10.1371/journal.pone.0006555)
Supplement: Figure S1 — Fifty-two candidate genes thought to be relevant to colon cancer by the new promoter structure algorithm were identified after initial analysis. A total of 50 genes were analyzed for detection of promoter methylation in CRC cell lines. UnM, unmethylated gene group; M, methylated gene group. (0.11 MB PPT) [file pone.0006555.s001.ppt]

## Slide 1
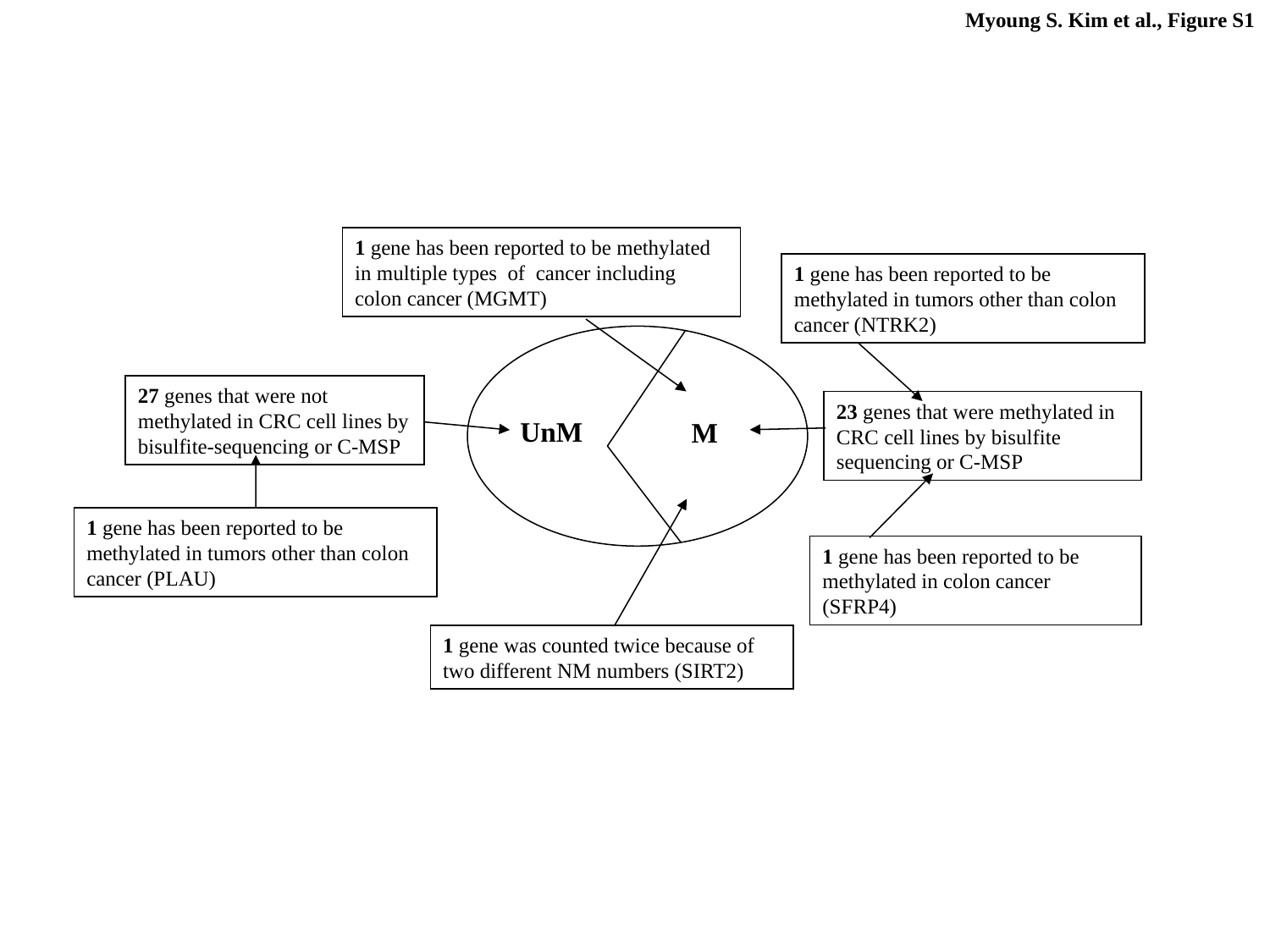

Myoung S. Kim et al., Figure S1
1 gene has been reported to be methylated in multiple types of cancer including colon cancer (MGMT)
1 gene has been reported to be methylated in tumors other than colon cancer (NTRK2)
27 genes that were not methylated in CRC cell lines by bisulfite-sequencing or C-MSP
23 genes that were methylated in CRC cell lines by bisulfite sequencing or C-MSP
UnM
M
1 gene has been reported to be methylated in tumors other than colon cancer (PLAU)
1 gene has been reported to be methylated in colon cancer (SFRP4)
1 gene was counted twice because of two different NM numbers (SIRT2)
