# Supplementary material for: Promoter DNA Methylation of Oncostatin M receptor-β as a Novel Diagnostic and Therapeutic Marker in Colon Cancer
Source: PLoS One. 2009 Aug 7;4(8):e6555. doi: 10.1371/journal.pone.0006555 (PMC2717211; doi:10.1371/journal.pone.0006555)
Supplement: Figure S2 — Representative results of bisulfite sequencing in CRC cell lines and tissues (A). Genomic and bisulfite-treated genomic DNA sequences are indicated. *, Cytosine that was not protected by methylation was thus converted to Thymidine after bisulfite treatment. Underlined CGs, methylated CpGs that were maintained after bisulfite treatment. Primer sequences for bisulfite-sequencing were described previously (12). B, Promoter methylation of TUBG2 in cell lines was examined by C-MSP (a), and in tissues was examined by COBRA after digestion of gel-extracted PCR products with BstU1 (b). Samples were loaded on a 10% acrylamide gel, stained with 1 X SYBR Green Gold (Invitrogen) and visualized under UV light. Multiple cleaved bands by BstU1 digestion were detected in PT samples (1–5) after gel separation, indicating the continued presence of protected CGCG sequences as a result of methylation, whereas no BstUI cleavage was found in any NN samples (6–10). Due to tissue heterogeneity, methylated and unmethylated alleles co-exist in PT samples so that uncleaved bands can be seen. Mock digestion (without BstU1) of PT samples resulted in the same uncleaved band as BstU1 digestion of the normal colon mucosa PCR product. PT, paired CRC; NN, normal colon epithelium from non-cancer patients. (c), Representative bisulfite-sequencing results of the TUBG2 promoter. Only methylated alleles were detected in all cell lines tested whereas methylated/unmethylated alleles coexisted in PT and PN tissues. The criteria to determine methylation in cell lines and tissues are described in Materials and Methods. (1.48 MB PPT) [file pone.0006555.s002.ppt]

## Slide 1
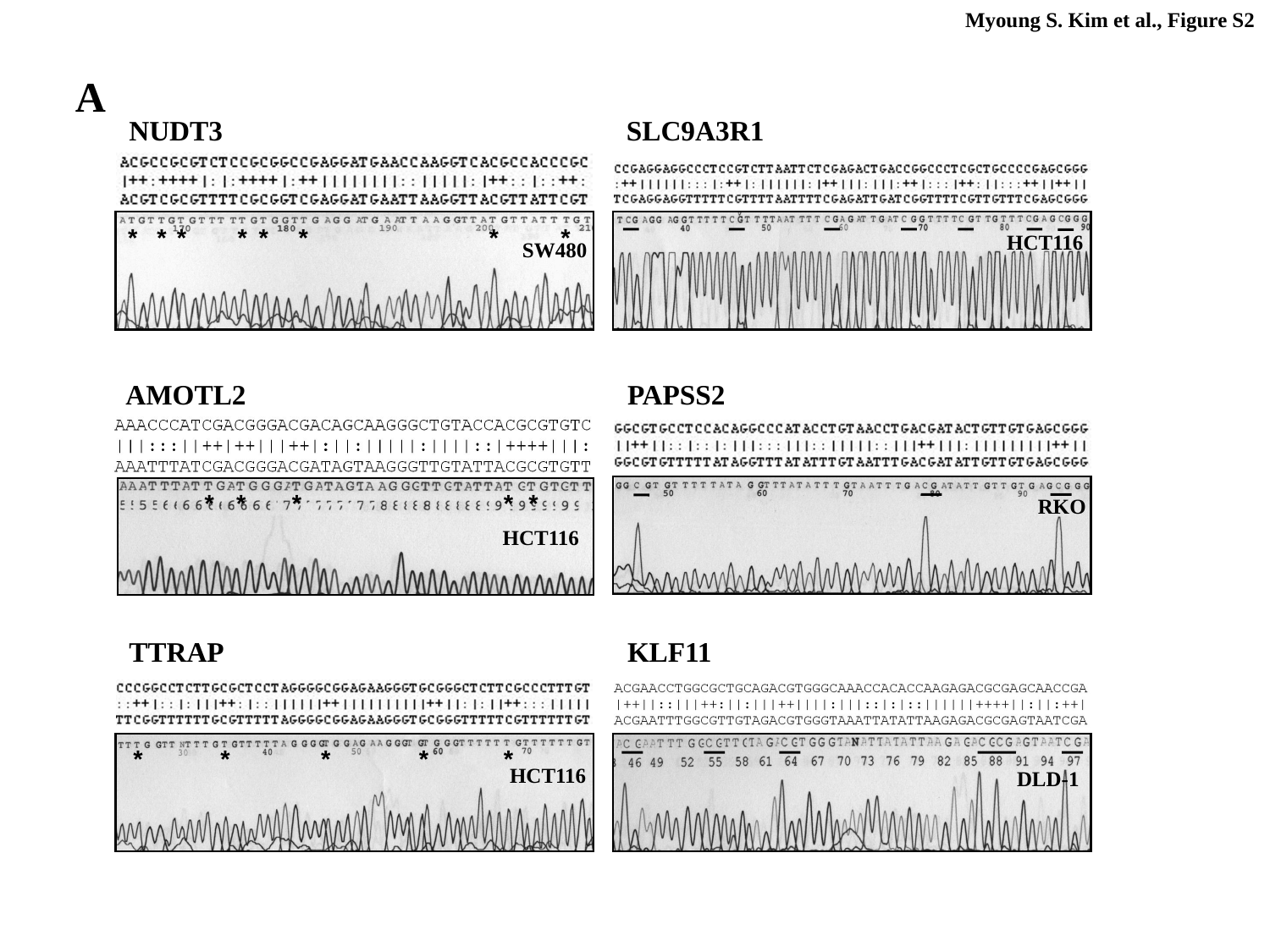

Myoung S. Kim et al., Figure S2
A
NUDT3
*
*
*
*
*
*
*
*
SW480
SLC9A3R1
HCT116
AMOTL2
HCT116
*
*
*
*
*
PAPSS2
RKO
TTRAP
*
*
*
*
*
HCT116
KLF11
DLD-1

## Slide 2
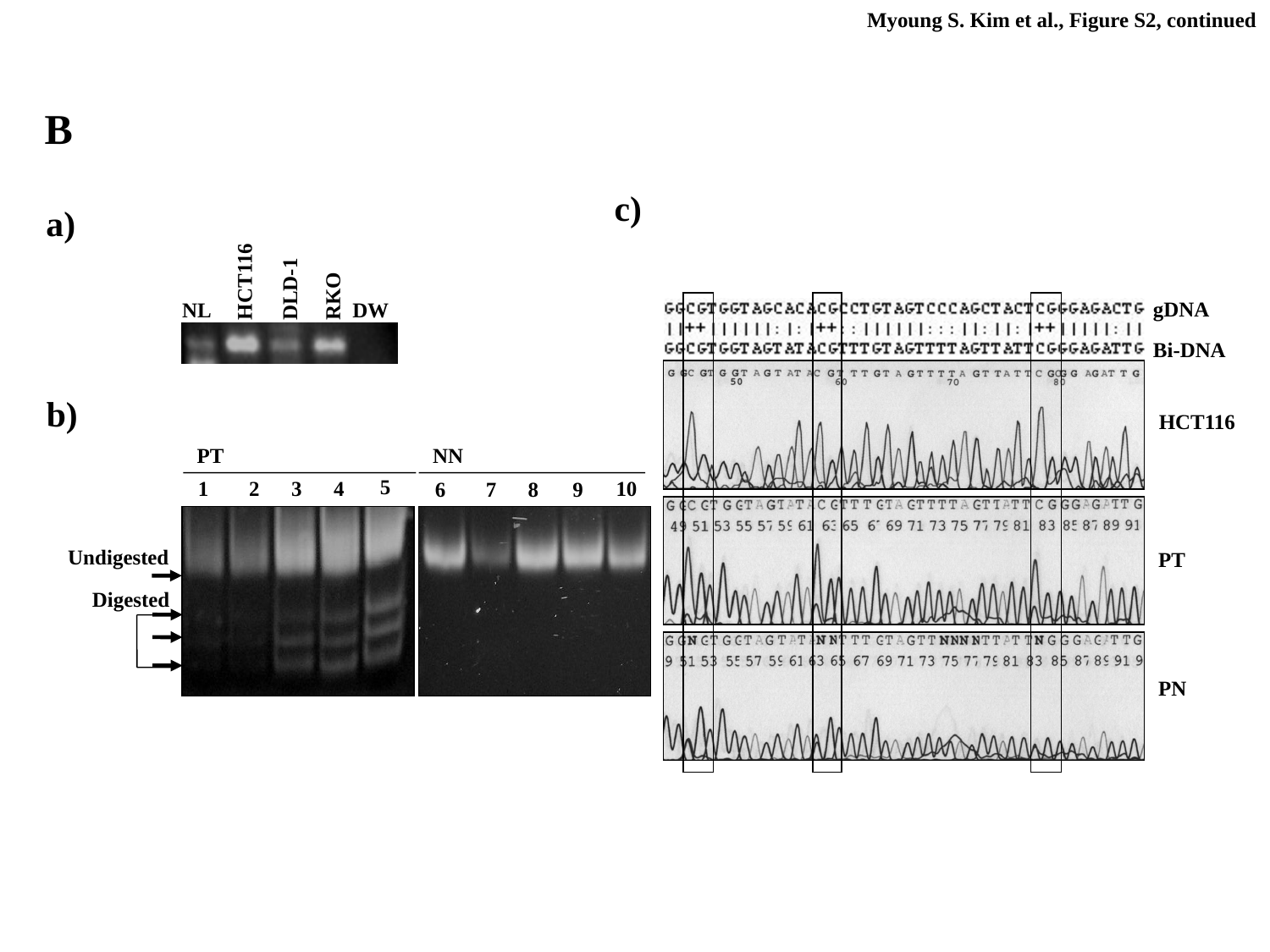

Myoung S. Kim et al., Figure S2, continued
B
c)
a)
HCT116
DLD-1
RKO
gDNA
Bi-DNA
HCT116
PT
PN
NL
DW
b)
PT
NN
5
1
2
4
3
10
6
7
9
8
Undigested
Digested
