# Supplementary material for: Promoter DNA Methylation of Oncostatin M receptor-β as a Novel Diagnostic and Therapeutic Marker in Colon Cancer
Source: PLoS One. 2009 Aug 7;4(8):e6555. doi: 10.1371/journal.pone.0006555 (PMC2717211; doi:10.1371/journal.pone.0006555)
Supplement: Figure S3 — CpG islands 1 Kb upstream of the transcription start site (TSS) in the candidate gene promoters. Graphics depicting CpG islands (gray) were taken from Methprimer software. NTRK2 and OSMR had two CpG islands and the other four genes had one CpG island within 1 Kb upstream of the TSS. Promoter regions analyzed in this study partially or completely covered the CpG islands indicated. Methylation for PAPSS2, TUBG2 and OSMR was examined at two different promoter regions. The location of the TSS is shown in each gene promoter. Primer sites for bisulfite-DNA amplification and C-MSP and TaqMan-MSP are indicated as Bi-F and Bi-R, and mF and mR, respectively. Probes for TaqMan-MSP analysis are indicated as P. Primers for C-MSP (mF-mR) were used for TapMan-MSP analysis. F, forward; R, reverse. (0.43 MB PPT) [file pone.0006555.s003.ppt]

## Slide 1
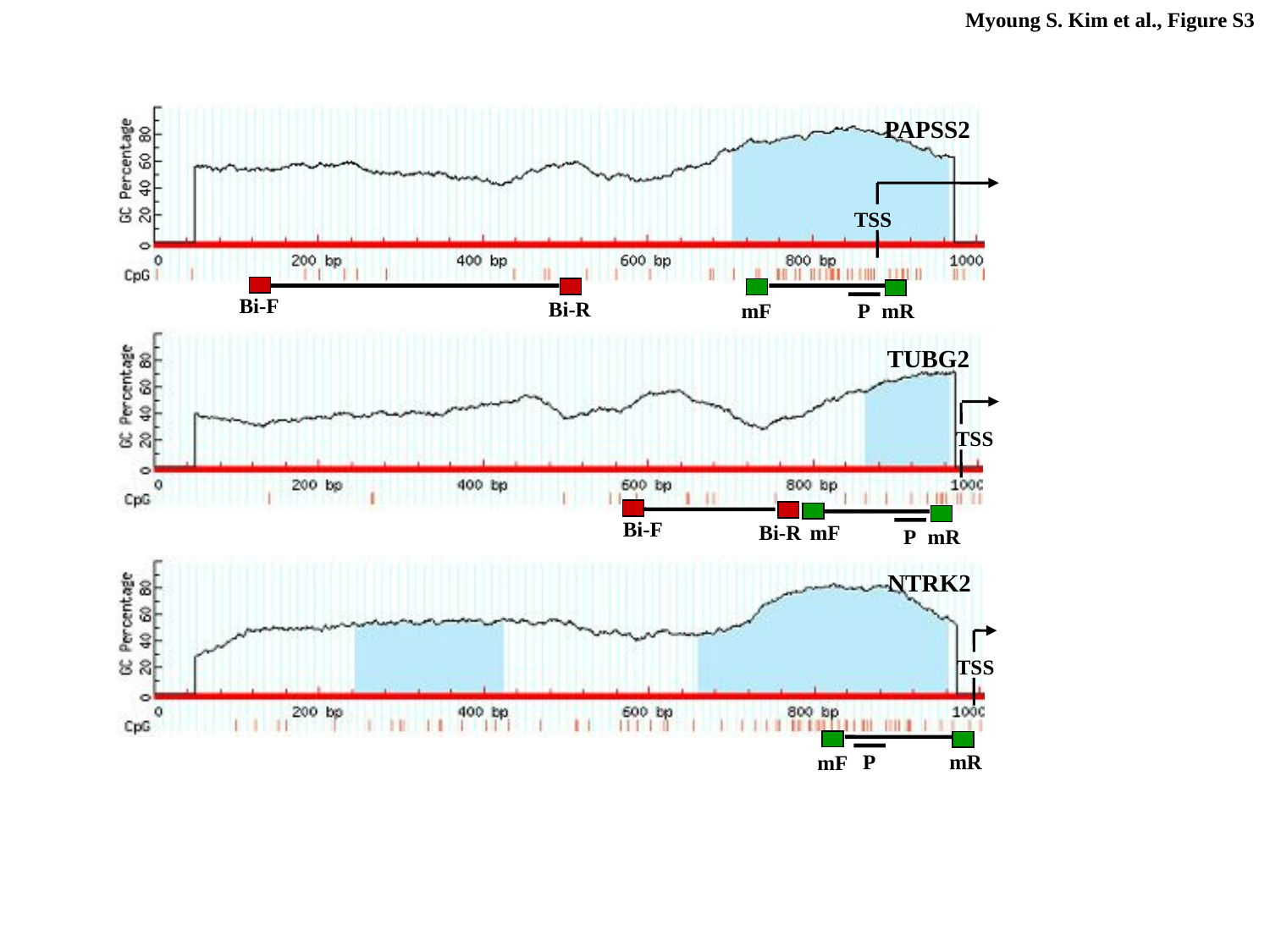

Myoung S. Kim et al., Figure S3
PAPSS2
TSS
Bi-F
Bi-R
mF
P
mR
TUBG2
TSS
Bi-F
Bi-R
mF
P
mR
NTRK2
TSS
P
mR
mF

## Slide 2
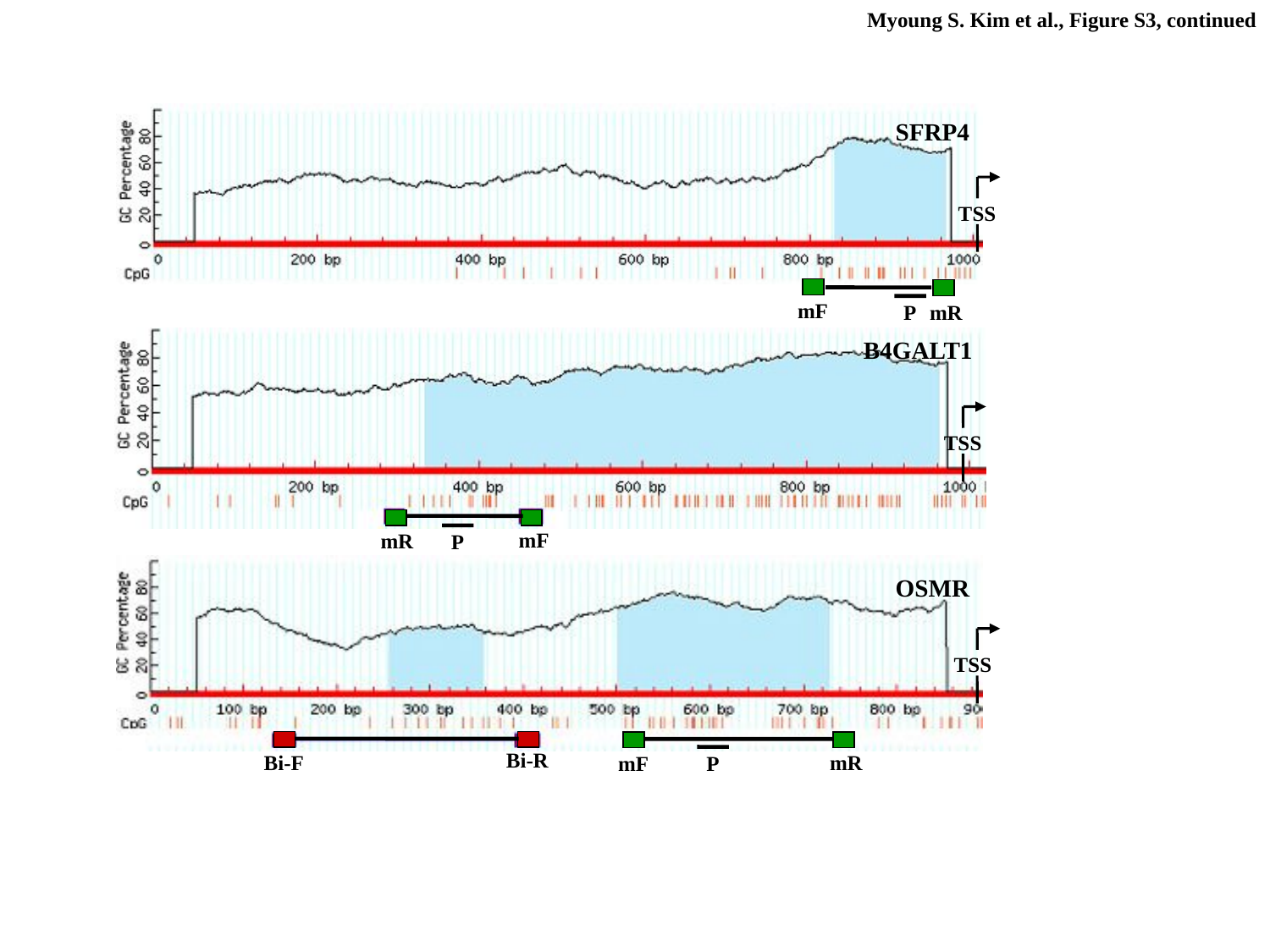

Myoung S. Kim et al., Figure S3, continued
SFRP4
TSS
mF
P
mR
B4GALT1
TSS
mF
mR
P
OSMR
TSS
Bi-R
Bi-F
mR
P
mF
