# Supplementary material for: Promoter DNA Methylation of Oncostatin M receptor-β as a Novel Diagnostic and Therapeutic Marker in Colon Cancer
Source: PLoS One. 2009 Aug 7;4(8):e6555. doi: 10.1371/journal.pone.0006555 (PMC2717211; doi:10.1371/journal.pone.0006555)
Supplement: Figure S4 — A, ROC curve analysis of 6 candidate genes (PT vs. NN). Area under the ROC (AUROC) conveys the accuracy in distinguishing NN from PT in terms of sensitivity and specificity. Solid line, genes analyzed; dashed line, no discrimination. B, ROC curve analysis (PT vs. PN) of B4GALT1 (left) and OSMR (right) in 100 pairs of CRC (PT) and corresponding normal (PN) tissues. (0.38 MB PPT) [file pone.0006555.s004.ppt]

## Slide 1
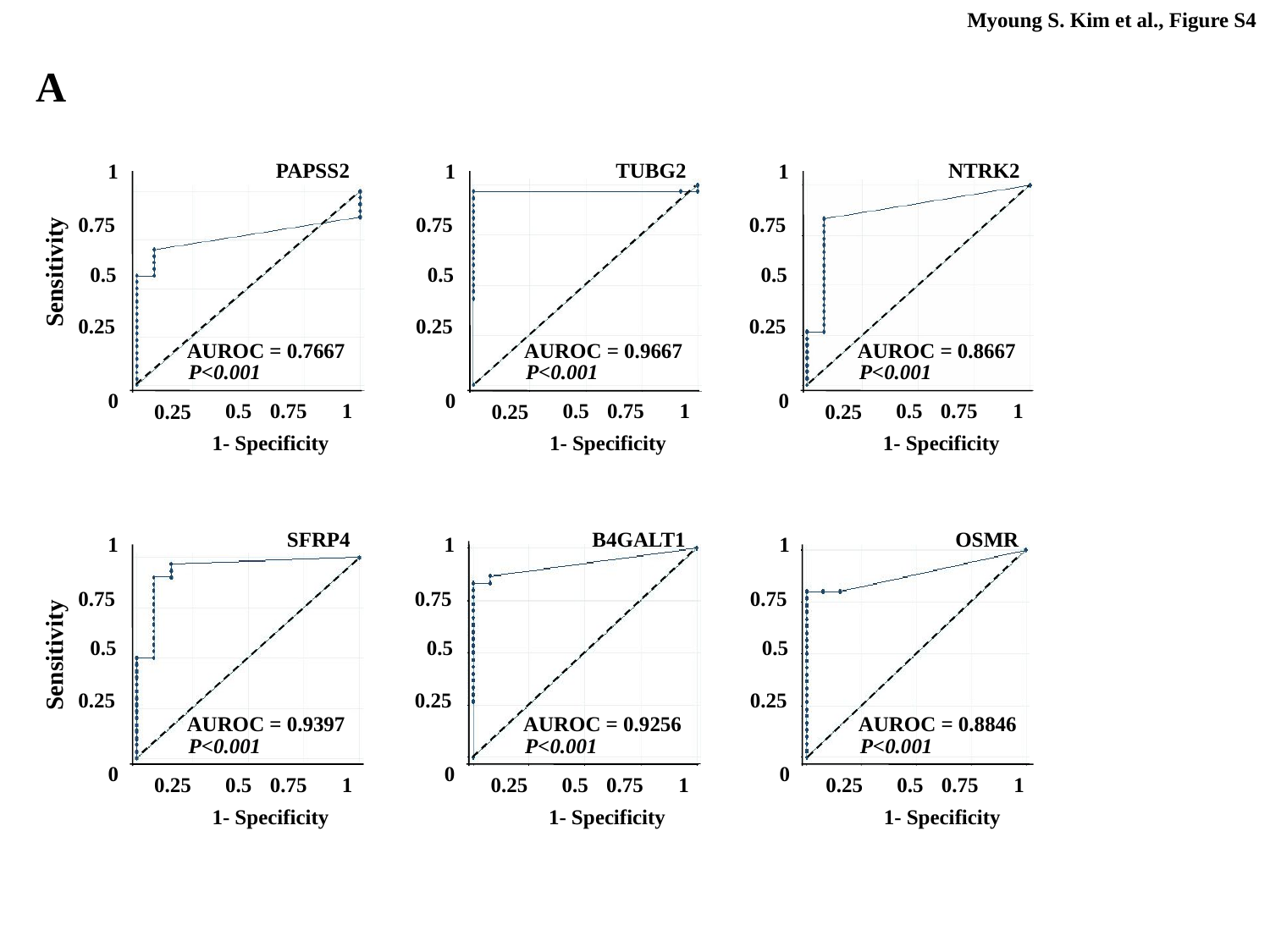

Myoung S. Kim et al., Figure S4
A
PAPSS2
TUBG2
NTRK2
1
1
1
0.75
0.75
0.75
Sensitivity
0.5
0.5
0.5
0.25
0.25
0.25
AUROC = 0.7667
AUROC = 0.9667
AUROC = 0.8667
P<0.001
P<0.001
P<0.001
0
0
0
0.5
0.75
1
0.5
0.75
1
0.5
0.75
1
0.25
0.25
0.25
1- Specificity
1- Specificity
1- Specificity
SFRP4
B4GALT1
OSMR
1
1
1
0.75
0.75
0.75
0.5
0.5
0.5
Sensitivity
0.25
0.25
0.25
AUROC = 0.9397
AUROC = 0.9256
AUROC = 0.8846
P<0.001
P<0.001
P<0.001
0
0
0
0.5
0.75
1
0.5
0.75
1
0.5
0.75
1
0.25
0.25
0.25
1- Specificity
1- Specificity
1- Specificity

## Slide 2
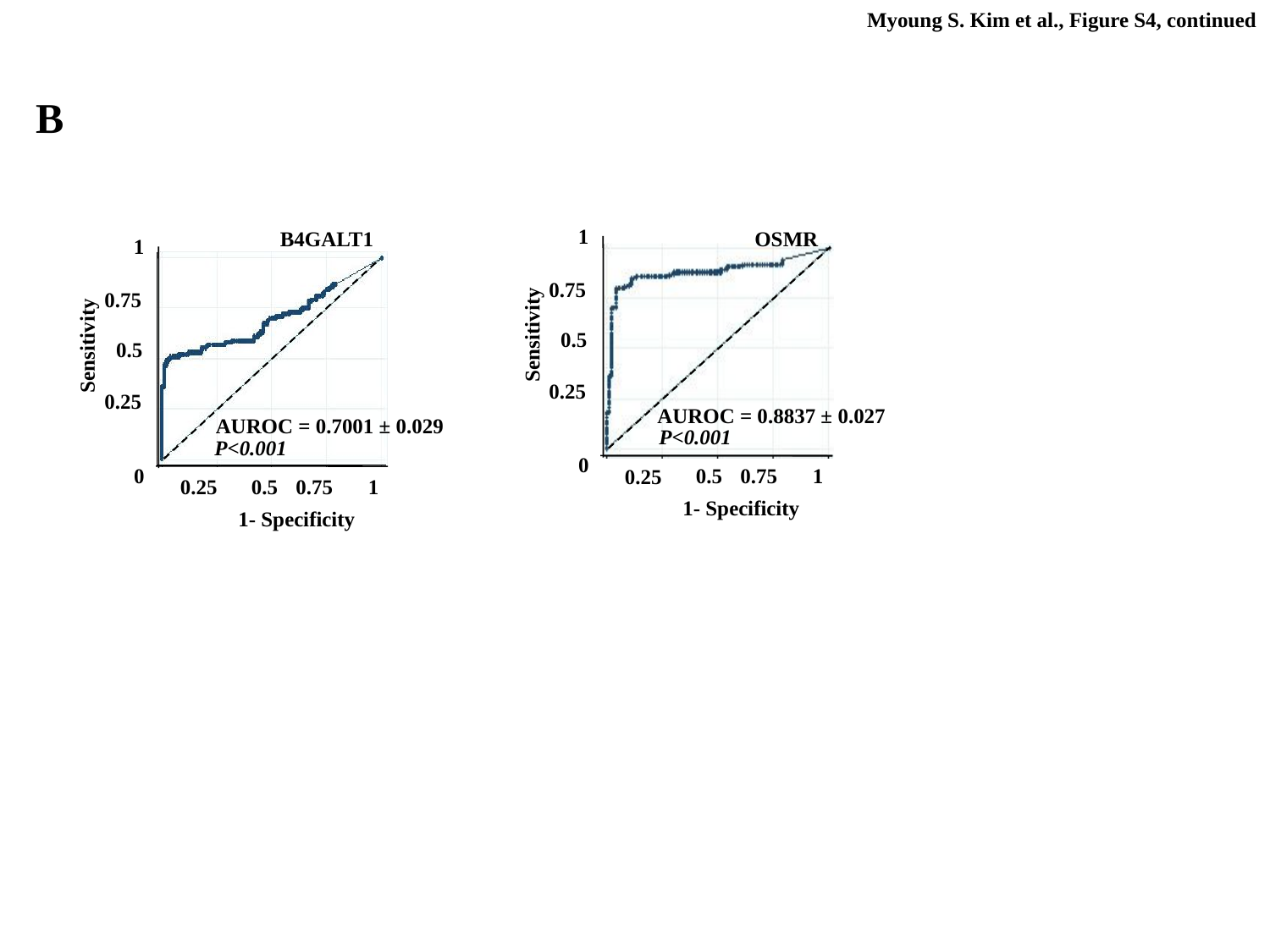

Myoung S. Kim et al., Figure S4, continued
B
1
B4GALT1
OSMR
1
0.75
0.75
Sensitivity
0.5
Sensitivity
0.5
0.25
0.25
AUROC = 0.8837 ± 0.027
AUROC = 0.7001 ± 0.029
P<0.001
P<0.001
0
0
0.5
0.75
1
0.25
0.5
0.75
1
0.25
1- Specificity
1- Specificity
