# Supplementary material for: Promoter DNA Methylation of Oncostatin M receptor-β as a Novel Diagnostic and Therapeutic Marker in Colon Cancer
Source: PLoS One. 2009 Aug 7;4(8):e6555. doi: 10.1371/journal.pone.0006555 (PMC2717211; doi:10.1371/journal.pone.0006555)
Supplement: Figure S5 — Expression of B4GALT1 and OSMR. A, B4GALT1 was reactivated by the 5-Aza-dC treatment (Aza), and OSMR reactivation by 5-Aza-dC was previously reported [12]. Fold induction was calculated by comparing the ratios of B4GALT1 or OSMR mRNA to GAPDH (an internal control) before and after treatment. Fold induction ranged from 1.7 to 4.4 for B4GALT1 and from 5.2 (HCT116) to 2,868 (DLD-1) for OSMR (12). Expression of the B4GALT1 (B) and the OSMR (C) in CRC cell lines was quantitatively compared by real-time RT-PCR. The lowest expression of OSMR was detected in SW480 cell line. Experiments were done in duplicate, and values indicate means±SD. *, P<0.05. (0.88 MB PPT) [file pone.0006555.s005.ppt]

## Slide 1
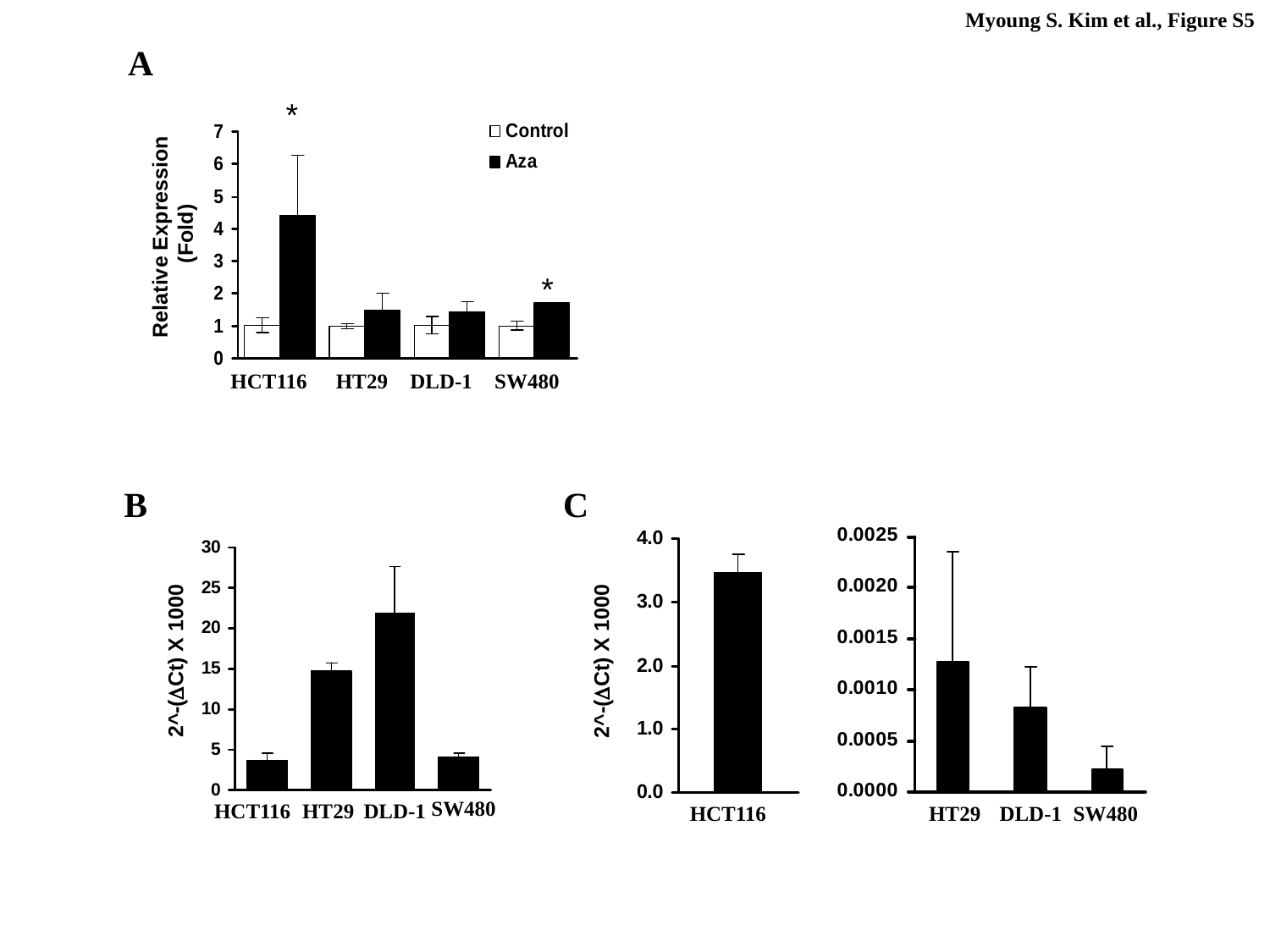

Myoung S. Kim et al., Figure S5
A
*
Relative Expression
(Fold)
*
HCT116
SW480
DLD-1
HT29
B
2^-(ΔCt) X 1000
C
2^-(ΔCt) X 1000
HCT116
HT29
DLD-1
SW480
SW480
HCT116
HT29
DLD-1
