# Supplementary figures and images for: Promoter DNA Methylation of Oncostatin M receptor-β as a Novel Diagnostic and Therapeutic Marker in Colon Cancer
Source: PLoS One. 2009 Aug 7;4(8):e6555. doi: 10.1371/journal.pone.0006555 (PMC2717211; doi:10.1371/journal.pone.0006555)

## Slide 1
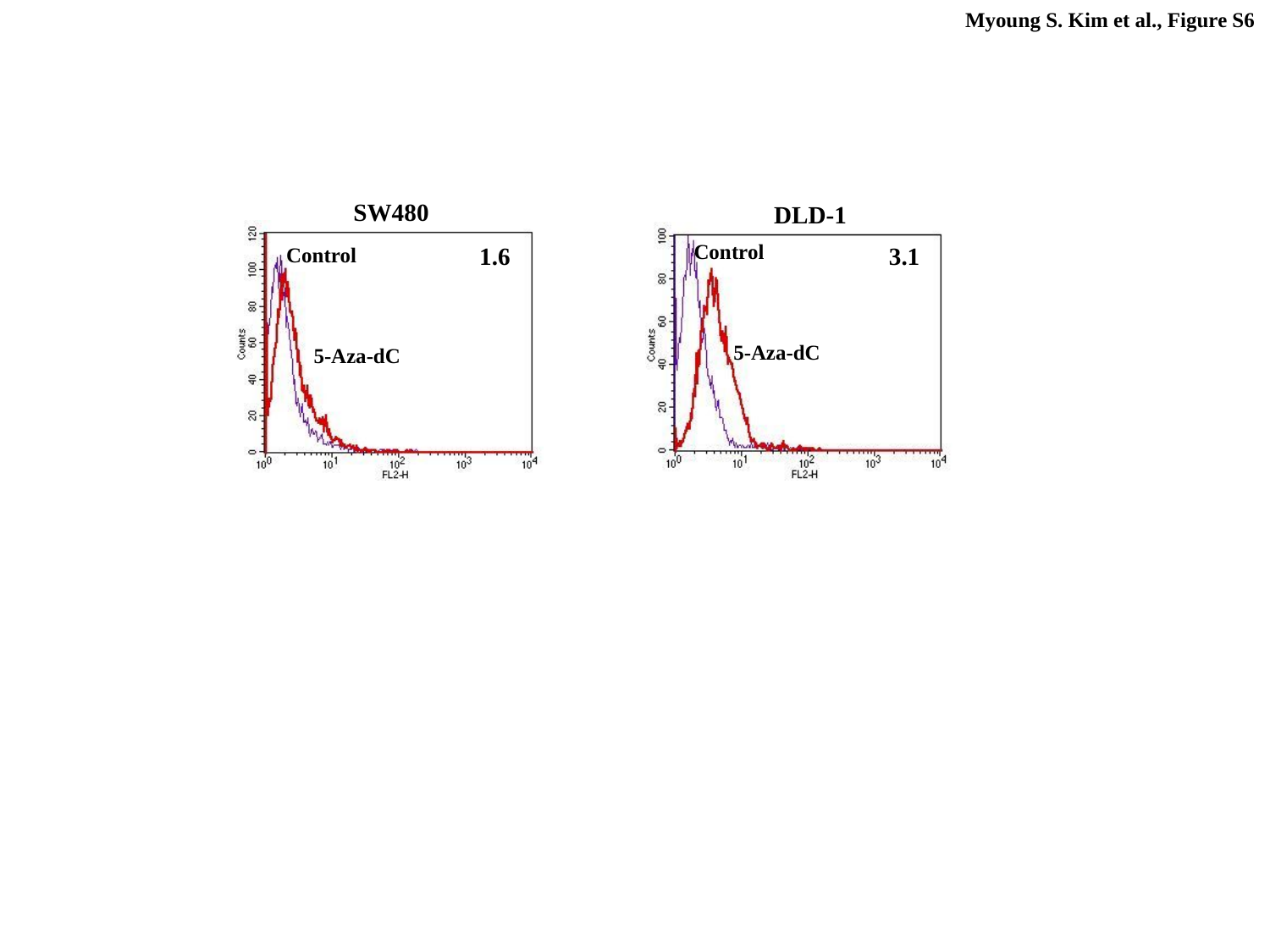

Myoung S. Kim et al., Figure S6
SW480
1.6
Control
5-Aza-dC
DLD-1
Control
3.1
5-Aza-dC

Supplement: Figure S6 — Re-activation of OSMR by 5-Aza-dC treatment. Cell surface expression of OSMR was determined on SW480 and DLD-1 cells by flow cytometry. Values shown as insets correspond to the mean of fluorescent intensity ratios between cells with or without 5-Aza-dC treatment (3 days). Mouse IgG-PE antibody was used as an isotype control (data not shown). (0.14 MB PPT) [file pone.0006555.s006.ppt]

## Slide 1
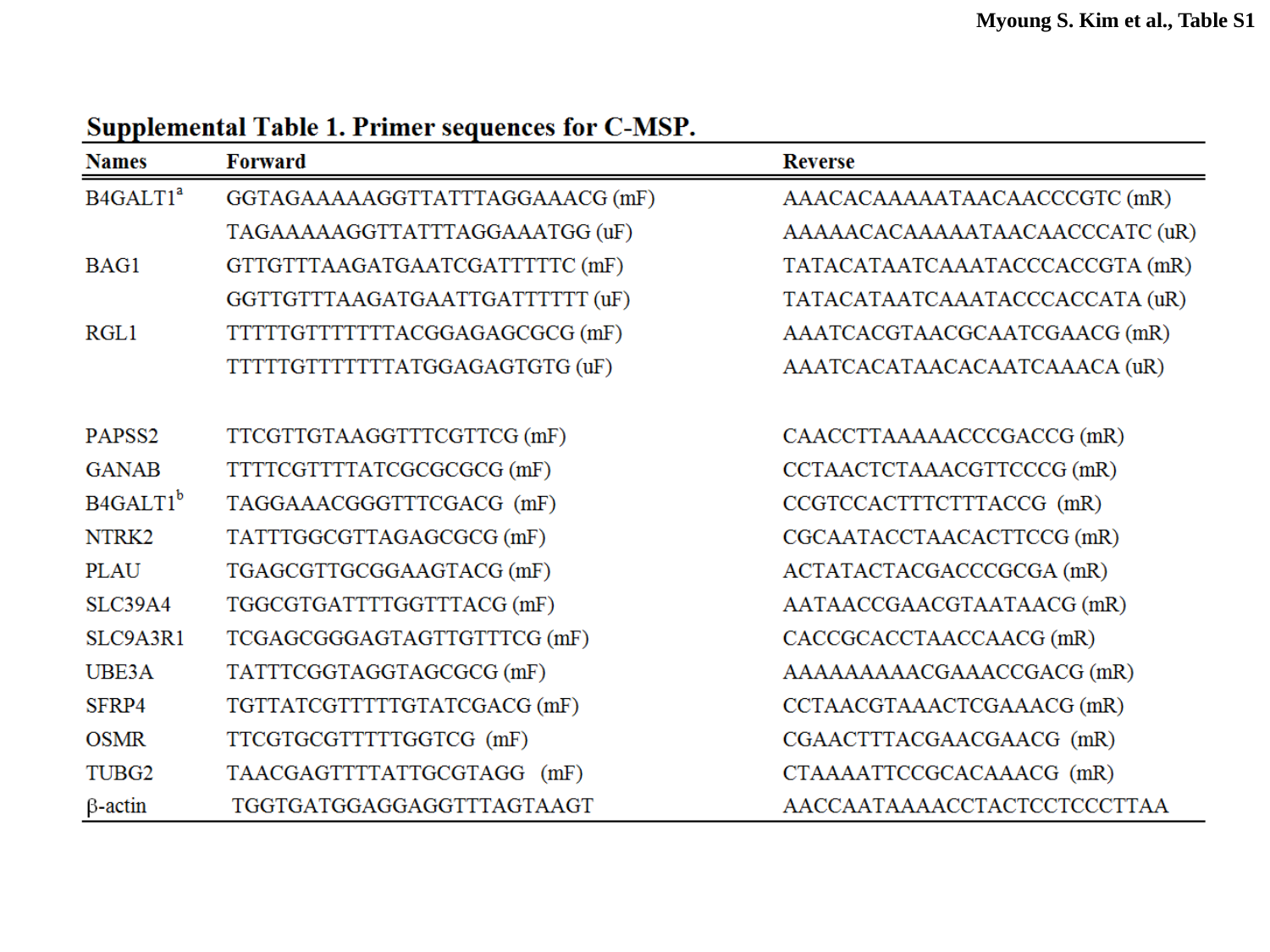

Myoung S. Kim et al., Table S1

Supplement: Table S1 — mF, methylation-specific forward; mR, methylation-specific reverse; uF, unmethylation-specific forward; uR, unmethylation-specific reverse. aPrimers used for detection of methylatoin in cell lines; bPimers in cell lines and tissues. (0.19 MB PPT) [file pone.0006555.s007.ppt]

## Slide 1
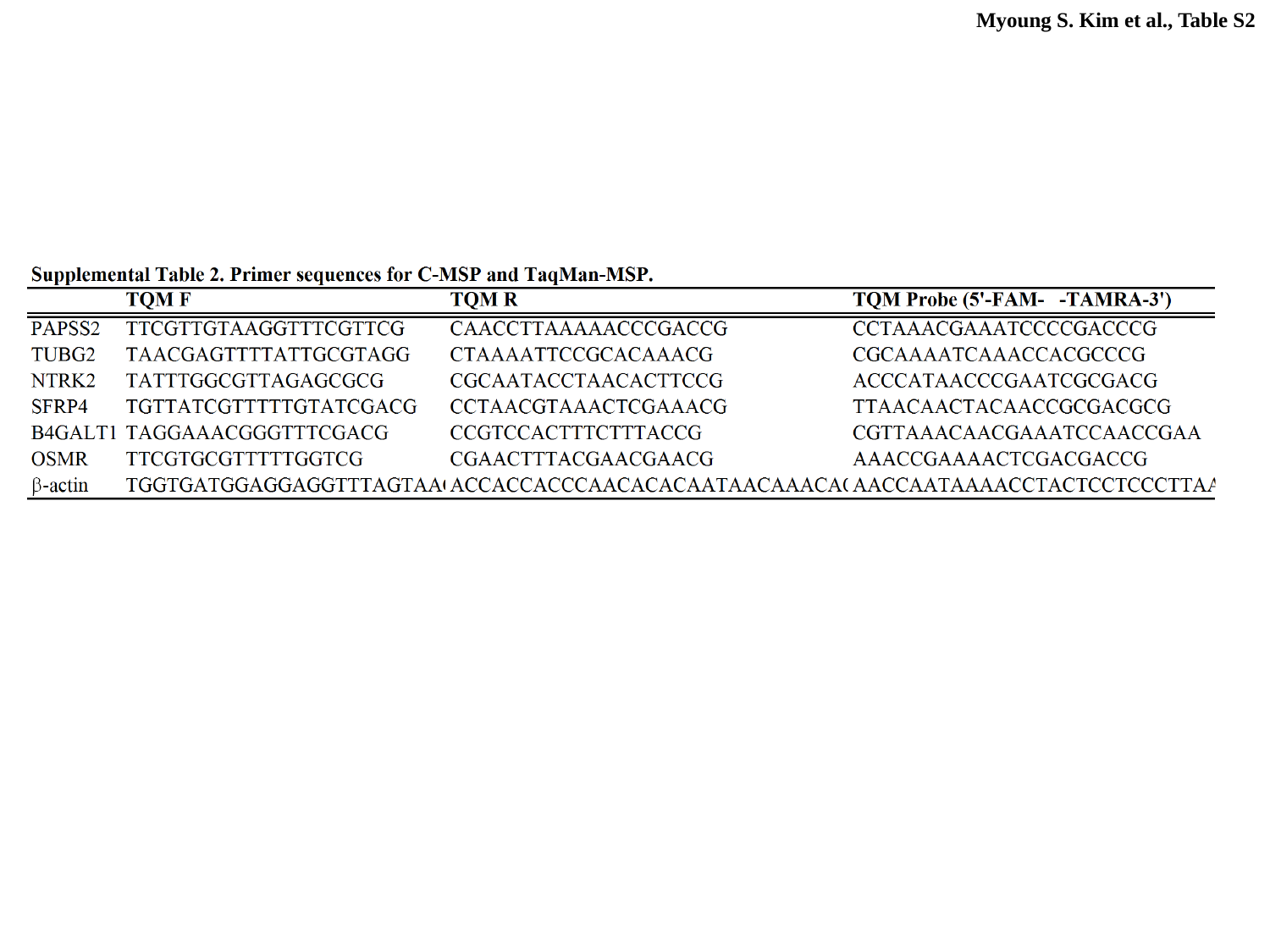

Myoung S. Kim et al., Table S2

Supplement: Table S2 — (0.17 MB PPT) [file pone.0006555.s008.ppt]

## Slide 1
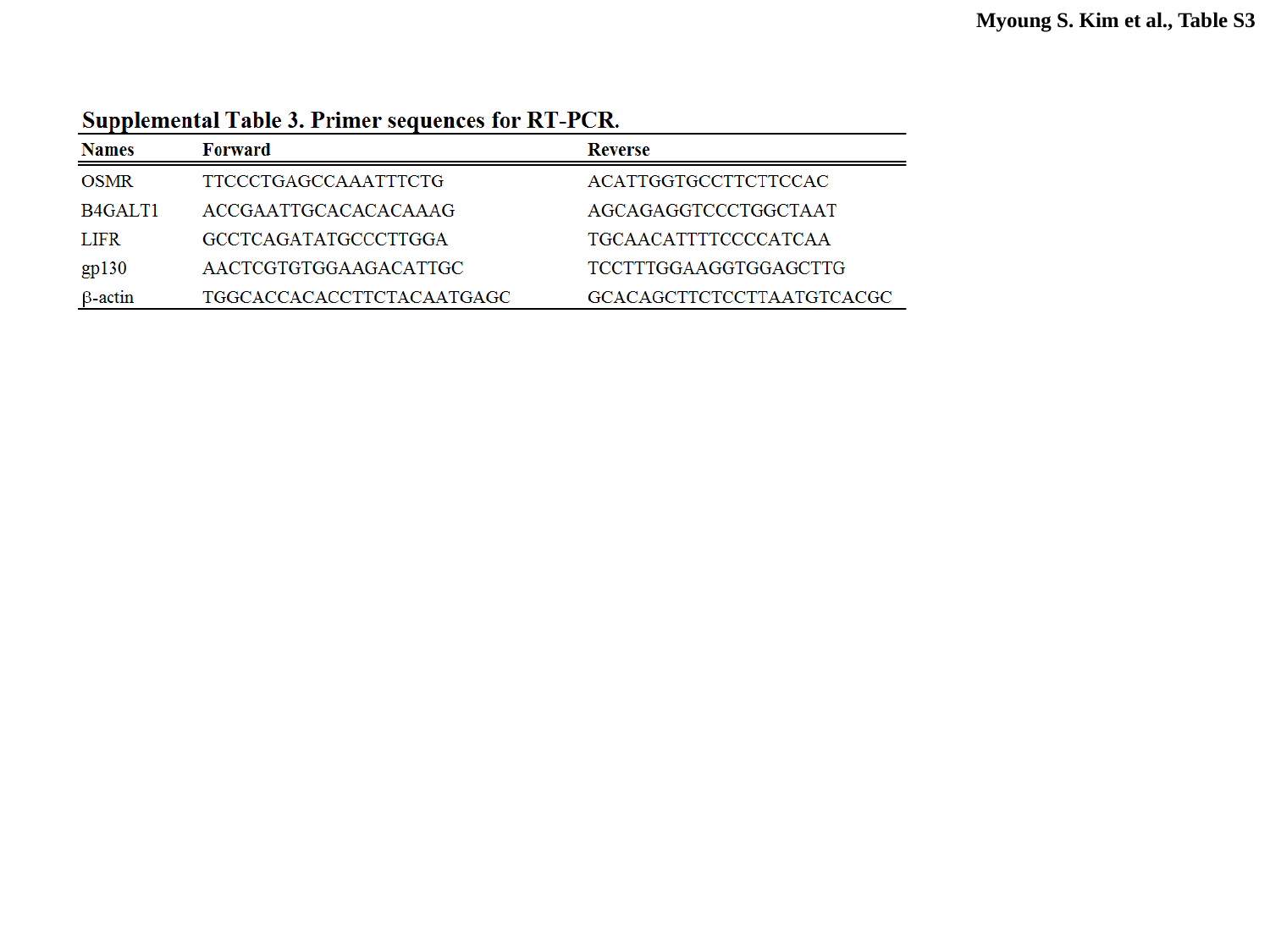

Myoung S. Kim et al., Table S3

Supplement: Table S3 — Primers for RT-PCR are the same as for Real-Time RT-PCR. (0.11 MB PPT) [file pone.0006555.s009.ppt]
